# Bioinspired Hemostatic Strategy via Pulse Ejections for Severe Bleeding Wounds

**DOI:** 10.34133/research.0150

**Published:** 2023-05-22

**Authors:** Bitao Lu, Enling Hu, Weiwei Ding, Wenyi Wang, Ruiqi Xie, Kun Yu, Fei Lu, Guangqian Lan, Fangyin Dai

**Affiliations:** ^1^ State Key Laboratory of Silkworm Genome Biology, College of Sericulture, Textile and Biomass Sciences, Southwest University, Chongqing 400715, China.; ^2^ Chongqing Engineering Research Center of Biomaterial Fiber and Modern Textile, Chongqing 400715, China.; ^3^ Division of Trauma and Surgical Intensive Care Unit, Research Institute of General Surgery, Jinling Hospital, Medical School of Nanjing University, Nanjing, 210002 Jiangsu Province, China.; ^4^ Department of Applied Biology and Chemical Technology, The Hong Kong Polytechnic University, Hong Kong, China.

## Abstract

Efficient hemostasis during emergency trauma with massive bleeding remains a critical challenge in prehospital settings. Thus, multiple hemostatic strategies are critical for treating large bleeding wounds. In this study, inspired by bombardier beetles to eject toxic spray for defense, a shape-memory aerogel with an aligned microchannel structure was proposed, employing thrombin-carrying microparticles loaded as a built-in engine to generate pulse ejections for enhanced drug permeation. Bioinspired aerogels, after contact with blood, can rapidly expand inside the wound, offering robust physical barrier blocking, sealing the bleeding wound, and generating a spontaneous local chemical reaction causing an explosive-like generation of CO_2_ microbubbles, which provide propulsion thrust to accelerate burst ejection from arrays of microchannels for deeper and faster drug diffusion. The ejection behavior, drug release kinetics, and permeation capacity were evaluated using a theoretical model and experimentally demonstrated. This novel aerogel showed remarkable hemostatic performance in severely bleeding wounds in a swine model and demonstrated good degradability and biocompatibility, displaying great potential for clinical application in humans.

## Introduction

Achieving high-performance hemostasis in complex and lethal wounds with massive bleeding remains a great challenge in natural disasters, traffic accidents, and battlefield settings [1]. The uncontrollable hemorrhage causes more than 30% of deaths worldwide and is the primary cause of severe morbidity and fatalities [2]. Various treatment modalities, including fibrin glue [3], gelatin/chitosan sponge [4,5], adhesive hydrogels [6], and hemostatic powders [7], have been widely developed to address these challenges. However, they are insufficient for complex wounds [8] because current hemostats cannot overcome harsh environments [9], including high-pressure blood flow, irregular wound shapes, and secluded bleeding sites, to reach deep bleeding sites. They can only promote coagulation on the wound surface, resulting in undesirable coagulation effects [10]. Therefore, an urgent need is to explore more efficient hemostatic materials, particularly for irregularly shaped wounds, deep and non-compressible bleeding wounds, and arterial bleeding wounds.

Blocking bleeding from the source via the systemic delivery of hemostats against blood flow using cargo-loaded micromotors or injectable volume-expansion sponges is a vital strategy for treating complex wounds. Recently, micromotors with specific actuation mechanisms (e.g., magnetic navigation or gas-induced propulsion) for the effective active delivery of hemostats to deep bleeding sites have shown great potential for use in complex wounds with irregular shapes [11,12]. For example, in our previous study [13], we designed thrombin-loaded colloidosomes that could actively release drugs via micro-ravines and enhance drug penetration inside the wound cavity through magnetic navigation and gas propulsion. However, the physical propulsion thrust of this material remains a challenge in massive bleeding wounds, especially in large animal bleeding wound models, because of its poor resistance to high-rate blood fluids and inflexible application approaches. Polymer-based volume-expansion sponges have been widely developed to treat non-compressible and deep massive bleeding wounds [14–16]. They can rapidly expand into the wound cavity upon contact with blood, forming a physical barrier that blocks deep bleeding sites.

Furthermore, these sponges, with excellent injectability and blood-triggered shape recovery capacity, can be directly delivered to the inside of the wound, and mechanical compression can be applied to the wound wall. Nevertheless, the most remarkable shortcoming is that volume-expansion sponges have difficulty filling the secluded and micro-ravines inside complex wounds owing to inadequate contact between sponges with regular geometric shapes and the small ravines inside the wound cavity [17]. An insufficient plug size may fail to achieve timely and efficient blood coagulation.

**Fig. 1.**
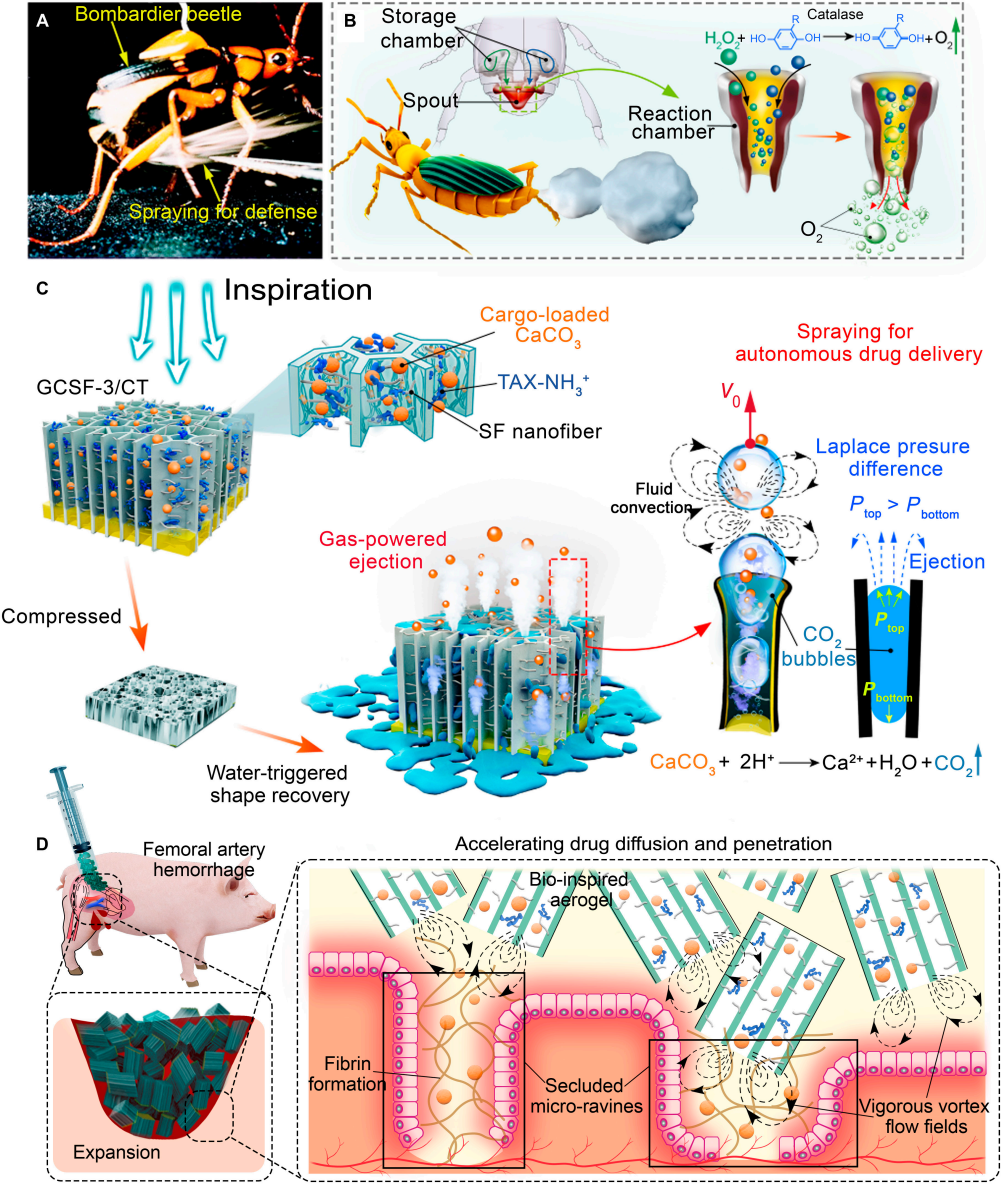
Fabrication and mechanism of the bombardier beetle-inspired aerogel. (A) Optical image of the bombardier beetle. Reproduced with permission [19]. Copyright 2005, Springer Nature. (B) Schematic illustration of pulse jet-like ejection performance of bombardier beetles. (C) The compressed bombardier beetle-inspired aerogels (GCSF-3/CT) with built-in cargo-loaded CaCO_3_ and protonated tranexamic acid (TXA-NH_3_^+^) when in contact with water/blood, leading to a shape recovery, gas-powered ejection, and enhanced cargo diffusion. *V*_0_, initial speeds; *P*_top_, Laplace pressure at the top; *P*_bottom,_ Laplace pressure at the bottom. (D) Hemostasis mechanism of bioinspired aerogel for severe bleeding wounds.

Bombardier beetles [18,19], an insect species of Coleopterans, can rapidly eject hot and toxic sprays from the tip of the abdomen at an ejection speed of approximately 10 m/s when threatened [20]. The mechanism of ejection from brachinine is based on the chemical reaction between catalases and peroxidases, which results in a large amount of oxygen in the reactive chamber, propelling toxic reaction products from the abdominal nozzles [21,22]. Such a biological “pulse jet” based on a chemical reaction could achieve a spray of several centimeters, approximately 4 times its body length, resulting in large-scale defense (Fig. 1A and B). Furthermore, the unique physiology of natural brachinine enabled us to construct a novel volume-expanding hemostatic aerogel compatible with the ejection system (Fig. 1C). Once in contact with blood, we hypothesized that such a brachinine-inspired aerogel could rapidly expand to eject gas microbubbles to promote hemostatic drug release, penetration, and diffusion to chemically activate blood coagulation and simultaneously recover to its original shape, acting as a barrier for physical blockage (Fig. 1D). This ejection performance mimics the defense behavior of brachinines, generating gas-induced efficient fluid convection and effectively accelerating drug penetration into small-sized ravines inside the wound cavity in cooperation with expanded aerogels to achieve highly adequate synergistic hemostasis via physical and chemical approaches. Therefore, incorporating a brachinine-inspired ejection system into a volume-expansion aerogel can create a hemostatic material with enhanced therapeutic efficiency.

The fabrication of a unique 3-dimensional (3D) structure that possesses a combination of properties involving a water/blood-triggered ejection system and shape-memory property is the priority to transition this bombardier beetle-inspired hemostatic aerogel from a proof-of-concept to a practical product. Directional freeze-casting [23] is a simple and versatile technique based on ice crystal templates that has received much attention for constructing various 3D aerogels with hollow and aligned microchannel structures [24]. Unlike sponges with random porous structures formed via the conventional ice-templating method, the parallel-aligned microchannels, similar to the vascular tissue structure of trees, could promote liquid transport with a higher absorption speed owing to their lower tortuosity and lower resistance [25]. Furthermore, parallel channels, which are promising platforms owing to their large interior spaces, can be loaded with various therapeutic cargos to endow structures with specific attributes. This technology has also been utilized to develop several aerogels with high-performance functions (e.g., shape memory properties [26], high strength [27], and thermal insulation performance [28]) by adjusting the assembly units and freeze-casting processes. Thus, a practical approach may be using directional freeze-casting to establish shape-memory aerogels equipped with brachinine-inspired ejection systems.

However, incorporating an ejection system into shape-memory hemostatic aerogels to mimic bombardier beetles has not yet been investigated. In this study, we report a novel material that assembles electrospun fibroin nanofibers and chitosan into blocky nanofibrous aerogels with aligned microchannel arrays via directional freeze-casting, followed by loading with cargo-carried CaCO_3_ and TXA-NH_3_^+^ through the vacuum loading method to obtain bombardier beetle-inspired aerogels (GCSF/CT). Upon contact with water and blood, the compressed bombardier beetle-inspired aerogels can rapidly absorb water/blood through the microchannel structure and recover their original shape to press the wound wall, forming a physical barrier for bleeding wounds. TXA-NH_3_^+^ instantaneously reacts with CaCO_3_ to trigger the rapid release of uncountable gaseous CO_2_ microbubbles within the microchannels. These microbubbles then accumulate gradually and burst to induce vigorous vortex flow fields, resulting in powerful and continuous pulse jet-like ejections, further accelerating drug diffusion and penetration into the wound cavities. Because of the synergistic effects of shape-recovery ability and ejection behavior, this bombardier beetle-inspired aerogel is anticipated to achieve highly adequate hemostasis, displaying great potential for clinical applications in the treatment of various complex wounds. Bleeding wounds were established over a swine model to prove the feasibility of this concept, which is very limited in biomaterial studies for hemostasis.

## Results and Discussion

### Preparation and characterization of GCSF/CT

Inspired by the biological “pulse jet” of bombardier beetles, we aimed to fabricate aerogels with a hierarchical architecture that can achieve water/blood-triggered rapid shape recovery and actively produce internal explosions to accelerate payload ejection from aerogel microchannels. The bombardier beetle-inspired hemostasis aerogel was designed based on 2 critical steps: (a) A nanofiber-reinforced aerogel with a parallel-arranged microchannel was constructed using a directional freezing ice-templating method for robust shape memory ability and fast absorption of water and blood. (b) The aligned microchannel, as a loading platform, was fully loaded with cargo-carrying CaCO_3_ microspheres and TXA-NH_3_^+^ to obtain an ejection system.

**Fig. 2.**
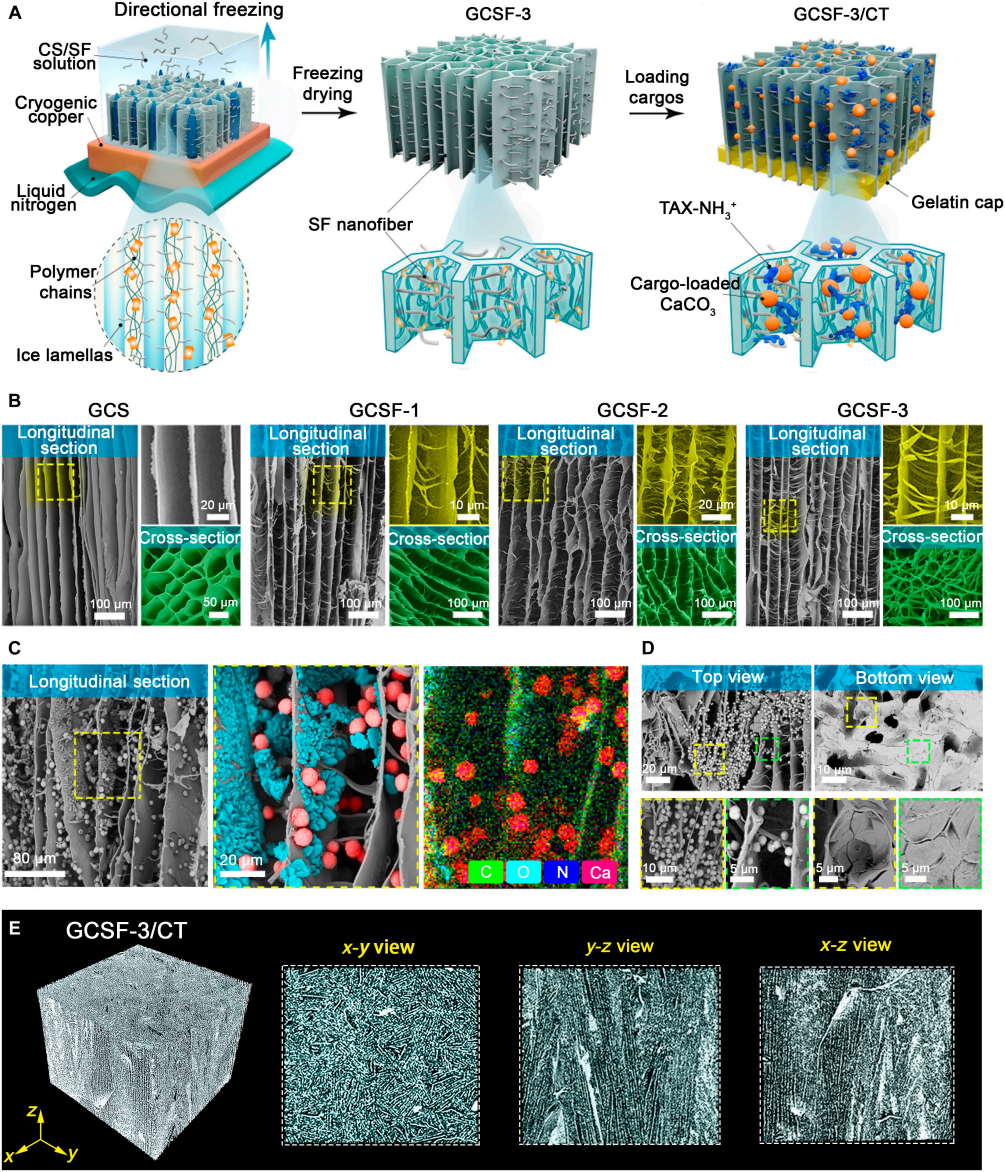
Preparation and characterization of GCS, GCSF-1/2/3, and GCSF-3/CT. (A) Directional freezing-assisted fabrication process of GCSF-3/CT. (B) SEM images illustrating the longitudinal section and cross-section of GCS and GCSF-1/2/3/CT. (C) Longitudinal section of GCSF-3/CT (the pink pseudocolor represents CaCO_3,_ and the blue pseudocolor represents TXA-NH_3_^+^) and corresponding mapping images. (D) Top-view and bottom-view images of GCSF-3/CT. (E) Micro-CT images of GCSF-3/CT.

The multicomponent GCSF/CT preparation procedure is summarized in Fig. 2A. Silk fibroin nanofibers, as the basic building block of GCSF/CT, was obtained by electrospinning, according to previous studies [29]. A flexible silk fibroin nanofiber membrane fabricated using electrospinning was crushed into a shortened nanofiber (S-NF) slurry under high-speed homogenization. The S-NF slurry and glutaraldehyde were added to a chitosan and acetic acid solution. After stirring, the mixture in the mold was placed on a cryogenic copper plate, directionally frozen, and lyophilized to obtain aerogels with different S-NF concentrations. In the freeze-casting process, ice crystals form at the bottom of the mixture under a temperature gradient, resulting in aerogels with multi-aligned microchannels. The S-NFs with feeding mass ratios 0%, 20%, 40%, and 60% were named GCS, GCSF-1, GCSF-2, and GCSF-3, respectively. As illustrated by the scanning electron microscopy (SEM) images of the longitudinal and cross-sectional views (Fig. 2B and Fig. S1A and B), the GCS aerogel possessed a hierarchical structure comprising parallel-arranged microchannels (with a width of 15 to 35 μm) and channel walls (with a thickness of approximately 0.8 μm). After introducing S-NFs, the SEM images of GCSF-1/2/3 illustrated that the structure of the aligned microchannel was inherited from the GCS. S-NFs (with a diameter of less than 1 μm) accumulated on the surface of the channel walls to form an interwoven fibrous network similar to the structure of the cuttlebone. The density of the fibrous network depended on the concentration of the S-NF slurry, possibly because the nanofibers were squeezed into the gaps between the ice crystals during the directional freezing process. In the subsequent lyophilization procedure, the sublimation of ice crystals led to the formation of parallel microchannels and the assembly of fibroin nanofibers with channel walls. Subsequently, heating further reinforced the crosslinking between the fibroin nanofibers and chitosan via glutaraldehyde to obtain aerogels with a hierarchical architecture.

GCSF-3 was selected as a representative loading platform further to explain the fabrication process of the brachinine-inspired hemostasis aerogel. The deposition of cargo-loaded CaCO_3_ and TXA-NH_3_^+^ followed the vacuum-loading method, and the gelatin cap end of the aerogel was obtained based on the infiltration process (Fig. 2A and Fig. S1C). As demonstrated in the SEM images of the longitudinal section, top view, and bottom view (Fig. 2C and D), cargo-loaded CaCO_3_ (red pseudocolor) and TXA-NH_3_^+^ (cyan pseudocolor) were distributed in the hollow microchannels. The elemental distributions (C, O, N, and Ca are shown in green, cyan, bluish violet, and red, respectively) further confirmed the successful deposition of cargo-loaded CaCO_3_ and TXA-NH_3_^+^. A gelatin cap was used to seal the one-sided pores of GCSF-3/CT to achieve highly efficient gas ejection and avoid premature leakage. As shown in Fig. 2D and Fig. S2A, the gelatin cap was labeled in pink pseudocolor, and the cover rate was greater than 80%, which can guarantee gas accumulation within microchannels. Furthermore, the micro-CT images confirmed the honeycomb-like porous structure in the *x*–*y* view and the aligned microchannel in the *y*–*z*/*y*–*x* view for GCSF-3 and GCSF-3/CT (Fig. 2E and Fig. S2B). These results indicated that the introduction of cargo-loaded CaCO_3_/TXA-NH_3_^+^ had no obvious effect on the pore structure. The cargo-loading capacity of GCSF-3/CT was approximately 48.6 ± 5.7 mg/cm^3^ (Fig. S1D). According to the thermogravimetric curve (Fig. S1F), the weight ratios of cargo-loaded CaCO_3_ and TXA-NH_3_^+^ were 11.48% ± 0.87% and 15.9% ± 0.49%, respectively. Figure 2E shows the porosities of the various aerogels based on micro-CT results. GCSF-3/CT showed a lower porosity (52% ± 2.5%) than GCS and GCSF-1/2/3 by introducing cargo-loaded CaCO_3_/TXA-NH_3_^+^ (Fig. S1E). In addition, all aerogels displayed outstanding hydrophilicity and hemophilicity, which may have contributed to the fast absorption of blood, thereby concentrating the clotting factors (Fig. S3A). Moreover, these aerogels can be processed into various shapes and dimensions owing to their unique physical properties (Fig. S3B).

### Water-triggered ejection from GCSF/CT and ejection mechanism

**Fig. 3.**
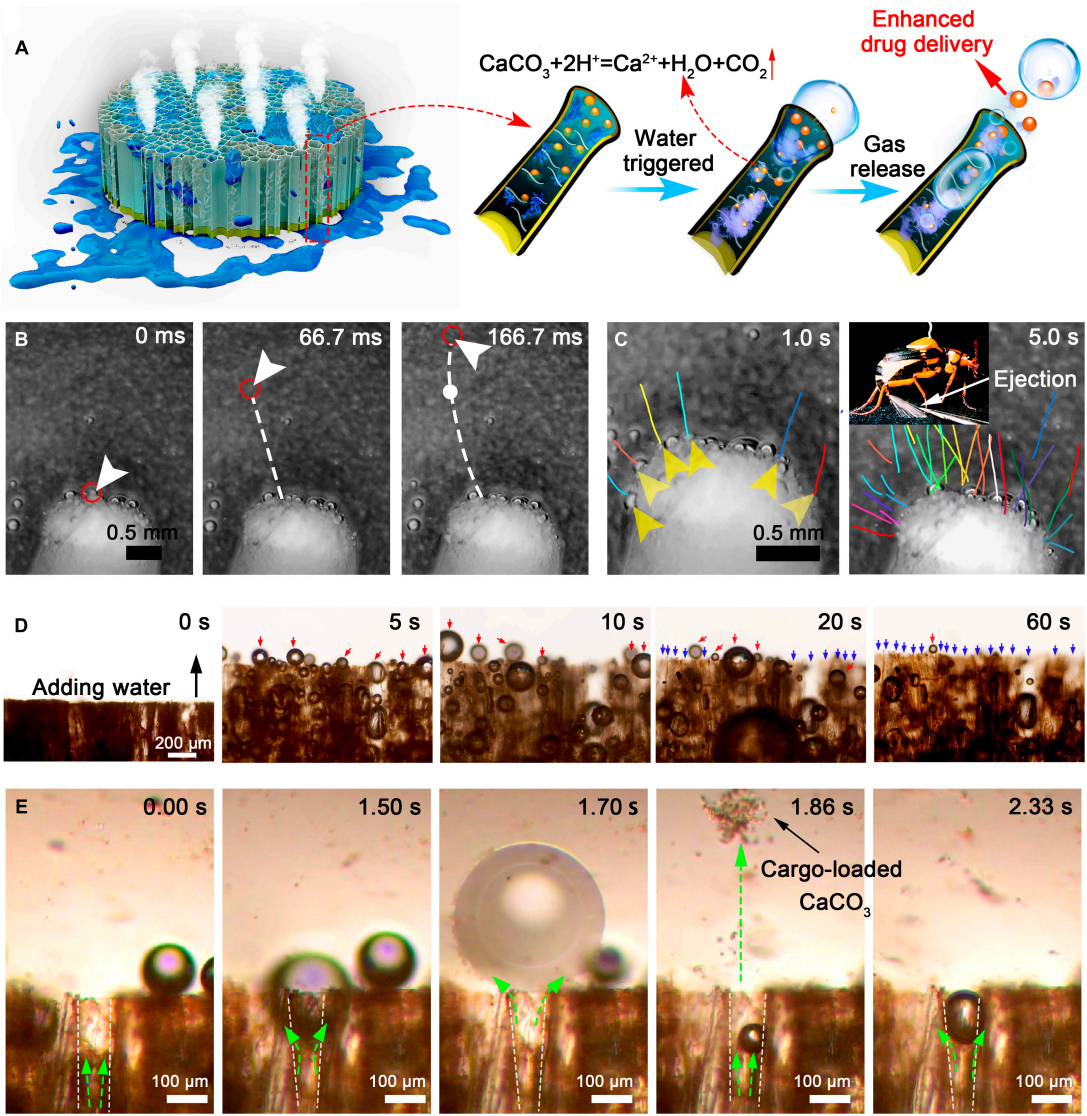
Water-triggered ejection behavior of GCSF-3/CT. (A) Schematic illustration of the ejection mechanism of bombardier beetle-inspired aerogels. (B) Images illustrating water-triggered rapid ejection from GCSF-3/CT under a high frame rate. (C) Time-frame images (0 to 5 s) depicting the displacement of microbubbles after contact with water under a high frame rate (these lines show the trajectories of microbubbles). (D) Microscopic images illustrating the gas-powered ejection performance and enhanced drug delivery of GCSF-3/CT after contact with water. The red and blue arrows indicate CO_2_ bubbles and empty microchannels, respectively. (E) Microscopy time-frame images taken from one microchannel showing the ejection of cargo-loaded CaCO_3_ under the propulsion of expanded microbubbles.

A schematic illustrating the ejection mechanism of the bombardier beetle-inspired aerogels is shown in Fig. 3A. The mechanism responsible for the ejection performance depends on the CO_2_ gas accumulation and expansion within the microchannels, causing a rapid, autonomous spray of payloads. GCSF-3/CT was selected as the representative aerogel to assess the ejection process in subsequent experiments. Movie S1, captured by a high-speed camera and corresponding time-frame images, corroborated the ejection trajectories (lines with various colors) of payloads from the microchannel of the aerogel in the Tween-20 solution within 5 s (Fig. 3B and C). A single microbubble was ejected about 1.98 mm within 166.7 ms (Fig. 3B) using one ejection trajectory as an example. In addition, the payloads were ejected in rapid pulses (>30 pulses within 5 s). The average speed of the ejected objects was 0.023 ± 0.0076 m/s (*n* = 4). The water-triggered ejection performance was further investigated using an inverted optical microscope. Figure 3D and Movie S2 show that the CO_2_ microbubbles rapidly expanded within the microchannel and were squeezed out of the aerogels upon contact with water, resulting in a propulsion thrust for accelerating drug diffusion. The ejection process could last for more than 60 s, and the ejection rate decreased with time. The aligned microchannels were almost empty after ejection, indicating a complete payload release. Additionally, Fig. 3E and Movie S3, under an optical microscope with higher magnification, clearly illustrate the ejection of cargo-loaded microparticles under the propulsion of expanded CO_2_ microbubbles. These results indicate that the entrapped cargo-loaded CaCO_3_/TXA-NH_3_^+^ significantly increased the delivery and diffusion of cargo via a gas-produced chemical reaction. Therefore, we concluded that the ejection system of aerogels, which highly simulates the physiological behavior of bombardier beetles, could serve as a built-in engine that favors the release and further permeation of cargo.

**Fig. 4.**
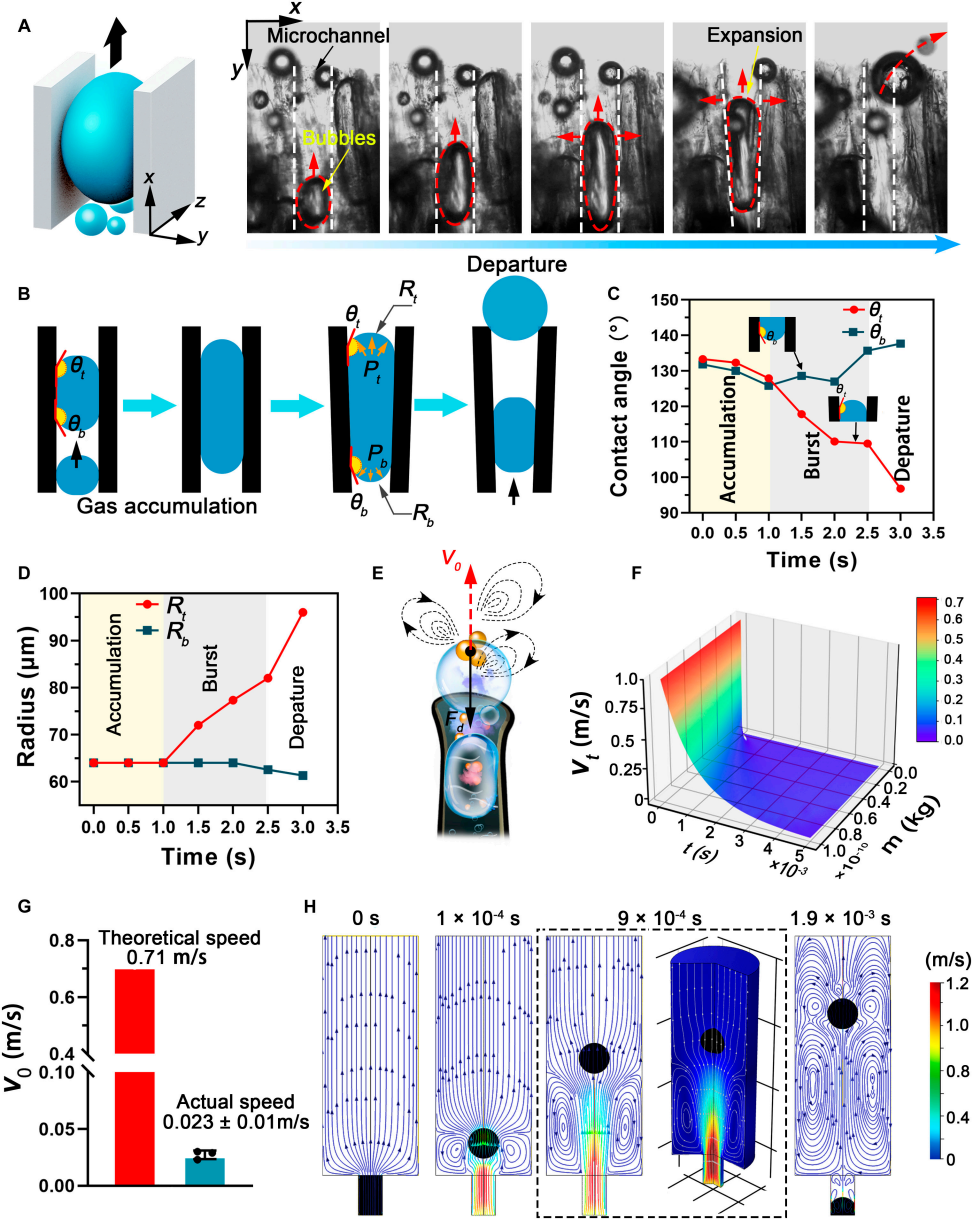
Mechanism and theoretical performance of microbubble ejection from GCSF-3/CT after contact with water. (A) A single microbubble ejected from the microchannel. (B) Schematic illustration of the microbubble ejection process. (C) The change of contact angles at the top (*θ_t_*) and bottom (*θ_b_*), and (D) the change of radius of curvature at the top (*R_t_*) and bottom (*R_b_*) during the microbubble ejection process. (E) Schematic of acting force on cargo-loaded microparticles under the propulsion of microbubbles. *F_d_*, drag force and gravity; *V*_0_, initial speeds. (F) Theoretical instantaneous speed profile of cargo-loaded microparticles after gas propulsion. (G) The maximum theoretical initial velocity and actual average speed of cargo-loaded microparticles. (H) COMSOL simulation of the ejection behavior and fluid convection generated from GCSF-3/CT after contact with water.

The CO_2_ microbubble propulsion and burst processes were observed and further investigated using a mathematical model to understand the mechanism of the ejection performance. As shown in Fig. 4A, a single microbubble with a growing volume migrated along the microchannel and was ejected, indicating an upward propulsion force for microbubble ejection. According to a previous study [30], the upward propulsion force is attributed to the difference in the Laplace pressure. As illustrated in Fig. 4B, in the initial stage, the microbubbles with a small volume exhibited a rounded rectangular shape with radial symmetry, that is, *θ_t_* = *θ_b_* and *R_t_ = R_b_*. During microbubble swelling in the microchannel, the contact angle *θ_t_* decreased, and the radius of curvature *R_t_* increased owing to the lateral expansion of the top microchannel walls under the pressure of expanding microbubbles.

In contrast, *θ_b_* and *R_b_* remained almost unchanged (Fig. 4C and D). Therefore, the continuous expansion of the microbubbles led to a steady increase in the difference between the radii of curvature of the top and bottom sides, resulting in an increased Laplace pressure difference (Δ*P_L_*). The Δ*P_L_* can be calculated as Eq. 1:

$$\Delta P_{L}\approx 2\gamma \;\left(\frac{1}{R_{b}}-\frac{1}{R_{t}}\right)\;$$ (1)

where γ is the surface tension of the water. Accordingly, Δ*P_L_* is inversely proportional to *R_b_* and directly proportional to *R_t_*. Therefore, when a microbubble reaches a critical size, the asymmetric pressure difference acts on the microbubble-triggered ejection, pushing payloads out of the microchannel through the opening.

A mathematical theoretical model was constructed to depict the gas-microbubble-powered induced ejection behavior further. For simplicity, we assumed that the spherical cargo-loaded microparticles maintained a constant radius (*R_s_*) during the drift, and the friction force was not considered in the calculation. When the CO_2_ microbubbles were ejected from the microchannel, the cargo-loaded microparticles were pushed by the force of microbubble propulsion and drifted at an initial speed. As illustrated in Fig. 4E, the drag force (*F*_*d*,_ including the liquid drag force and gravity) was mainly responsible for the movement of the payloads. According to Stokes’ law (Eq. 2), the drag force is proportional to the object’s speed in the solution. The instantaneous speed (*v_t_*) and initial speed (*v*_0_) were calculated using Eqs. 3 and 4, respectively.

$$F_{d}=kv_{t}=6\pi \eta R_{s}v_{t}$$ (2)

$$v_{t}=v_{0}e^{-\mathrm{kt}/m}$$ (3)

$$v_{0}=\frac{k∆x}{m\left(1-e^{-\mathrm{kt}/m}\right)}$$ (4)

where *k, t, m,* and ∆*x* are the resistance coefficient, time, payload mass, and payload displacement over time, respectively. As shown in Movie S1, the average initial speed of the cargo-loaded microparticles was estimated to be 0.023 m/s. The value of the maximum theoretical initial velocity was approximately 0.72 m/s, which was significantly higher than the actual speed owing to technological limitations in capturing the track of the microparticles (Fig. 4G). The *v_t_*–*m*–*t* profile was theoretically modeled based on Eqs. 3 and 4. As shown in Fig. 4F, the initial speeds were inversely proportional to the mass of the CMs, and the instantaneous speed of the CMs decreased rapidly over time.

A COMSOL Multiphysics simulation also corroborated the microbubble-induced ejection of payloads. As shown in Movie S4 and the corresponding time-lapse images in Fig. 4H, the microbubbles migrated from the bottom to the top, resulting in a pumping effect in the solution. The speed decreases with increasing displacement, which is consistent with the results of the theoretical model. Moreover, pump-like ejection produces autonomously active convective fluid flows, significantly increasing the permeation and diffusion of payloads. Based on the results mentioned above, the multicomponent aerogel inspired by bombardier beetles, as a promising active delivery system, can accelerate the permeation and diffusion of the payload within the bleeding site, resulting in increased contact between the hemostats and bleeding sites for rapid hemostasis.

### Shape-recovery ability, mechanical properties, and liquid absorbability of GCSF/CT

**Fig. 5.**
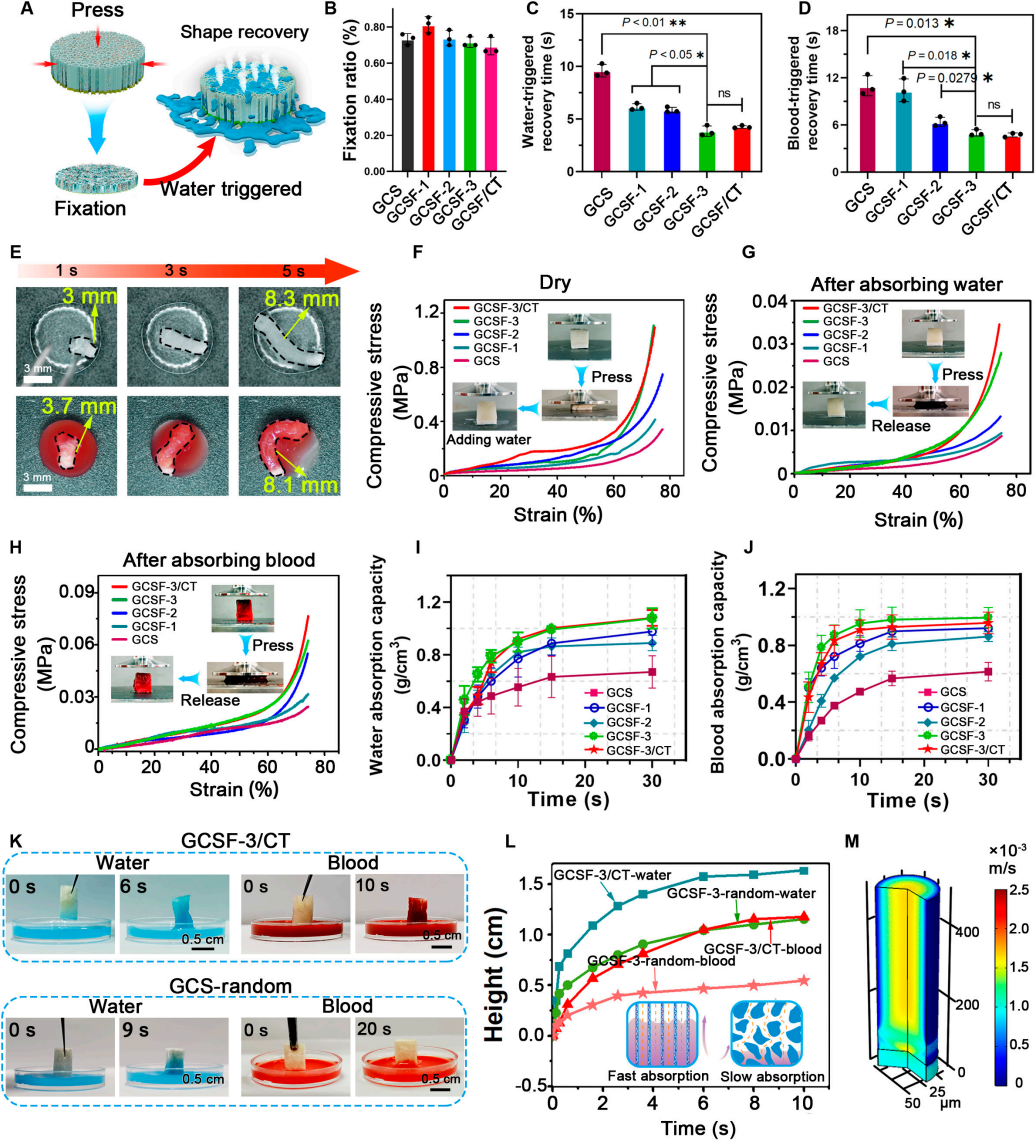
Shape-recovery ability, mechanical properties, and water/blood absorbability of various aerogels. (A) Schematic diagram of water-triggered shape recovery of GCSF-3/CT. (B) Fixation ratio of different aerogels. (C and D) The recovery time of compressed aerogels after contacting water and blood. (E) Optical images of water/blood-triggered shape recovery of GCSF-3/CT. (F) Compressive stress–strain curves of various aerogels in the dry state. (G and H) Compressive stress–strain curves of various aerogels before and after absorbing water/blood. (I and J) Water/blood absorption capacity of compressed aerogels. (K and L) Macro photographs illustrating the water/blood absorbability of aerogels with different porous structures. (M) COMSOL simulation images of liquid absorbability.

The scheme in Fig. 5A shows that bombardier beetle-inspired aerogels with fixed shapes can restore their initial shapes and generate gas-powered propulsion after contact with water and blood. The combination of mechanical compression at the bleeding site and ejection performance was responsible for the bombardier beetle-inspired aerogel mechanism of rapid hemostasis. Therefore, evaluation of the water/blood-triggered volume expansion performance, mechanical properties, and liquid absorption ability is essential for further applications of GCSF/CT. Figure 5B shows that the fixed ratio for all aerogels was greater than 80%. After absorbing water/blood, the shape-fixed GCSF-3/CT rapidly absorbed water/blood and restored its original shape with a recovery ratio of approximately 100% (Fig. S4A and B). The recovery time of GCSF-3/CT (4.24 ± 0.1 s) was shorter than that of GCS and GCSF-1/2, similar to that of GCSF-3 (Fig. 5C). Even in fresh blood, GCSF-3/CT could achieve full shape recovery within 4.74 ± 0.21 s (Fig. 5D and E).

Moreover, the recovery time after absorbing water and blood gradually decreased with increasing N-SFs. This phenomenon was ascribed to the interwoven fibrous nanofibers within the microchannels changing from a relaxed state to a bent state to generate elastic potential energy inside the aerogels during compression. The higher the number of nanofibers, the higher the elastic potential energy inside the compressed aerogels, resulting in the rapid recovery of GCSF-3/CT with water/blood inflow assistance. Interestingly, most previous studies demonstrated that hemostatic aerogels in blood take longer to recover their shape owing to their high blood viscosity [31]. However, in our study, the recovery times of GCSF-3/CT after contact with water and blood showed no evident differences. This was mainly ascribed to the aligned microchannels generating a strong capillary effect that promoted the penetration of blood into the GCSF-3/CT interior.

Volume-expansion aerogels should possess appropriate mechanical properties to avoid structural collapse caused by external stress or secondary bleeding due to over-extrusion, a severe concern for hemostasis. Figure 5F shows the compressive stress–strain curves of various aerogels in the dry state. With an increase in the S-NF concentration from 0 to 40 mg/ml, the compressive stress of aerogels under a 75% strain increased from 0.18 to 0.7 MPa (Fig. 5F), implying that the incorporation of silk fibroin nanofibers strengthened the network of aerogel. After introducing cargo-loaded CaCO_3_ and TXA-NH_3_^+^, the compressive stress of GCSF-3/CT showed no significant change compared with that of GCSF-3. After absorbing water and blood, the compressive stress of various aerogels was measured. As shown in Fig. 5G and H, the stress of all aerogels after absorbing water/blood significantly decreased compared with that in the dry state. In the water-treated group, GCSF-3 and GCSF-3/CT exhibited superior compressive mechanical performances, with compressive stresses of 0.023 and 0.02 MPa, respectively.

In contrast, GCSF-3 and GCSF-3/CT exhibited enhanced mechanical strength after absorbing blood, owing to the formation of blood clots within the microchannels of the aerogel. In addition, after 10 cycles of repeated compression, the GCSF-3/CT in the wet state did not collapse/fracture and only exhibited a 5% plastic deformation. The Young’s modulus and mass stress remained stable (Fig. S4D), indicating good reusability and durable fatigue resistance (Fig. S4C). Meanwhile, the storage modulus, loss modulus, and damping rate of GCSF-3/CT under a 75% strain remained constant over the angular frequency range from 0.1 to 100 Hz, indicating good elastic response performance (Fig. S4E). The water and blood absorbability of the aerogels were also characterized (Fig. 5I and G). The maximal water/blood absorption ratios of the GCSF-3/CT and GCSF groups showed no evident differences and were higher than those of the other groups. The water/blood absorption capacity gradually increased with increasing N-SF content, owing to the increased surface area of the microchannels. In addition, the higher density of the nanofiber network in the microchannels resulted in a significantly higher retention ability. Therefore, GCSF-3/CT possessed remarkable liquid absorbability, which allowed water to diffuse into the inner microchannels to trigger injection behavior rapidly. To further confirm the liquid absorption of GCSF-3/CT, aerogels with a random pore structure (GCSF-3-random) were prepared for comparison. The height of the water/blood absorbed into the aerogels versus time is shown in Fig. 5K and L.

In contrast, the liquid absorption speed of GCSF-3/CT was superior to that of GCSF-3-random, similar to the results of a previous study [25] because the aerogel with an aligned microchannel could decrease energy consumption and flow resistance, leading to fast water/blood absorbability. We further illustrated the liquid absorption performance of GCSF-3/CT using COMSOL (Fig. 5M and Movie S5). The parameters were set based on the SEM images, and the model was tested using a laminar flow model. The liquid flow speed in the aligned microchannel could reach 1.5 × 10^−3^ m/s. This theoretical model further corroborated that GCSF-3/CT with aligned microchannels could effectively accelerate the penetration of water and blood, implying the enormous potential for hemostasis.

### Description of cargo release performance

**Fig. 6.**
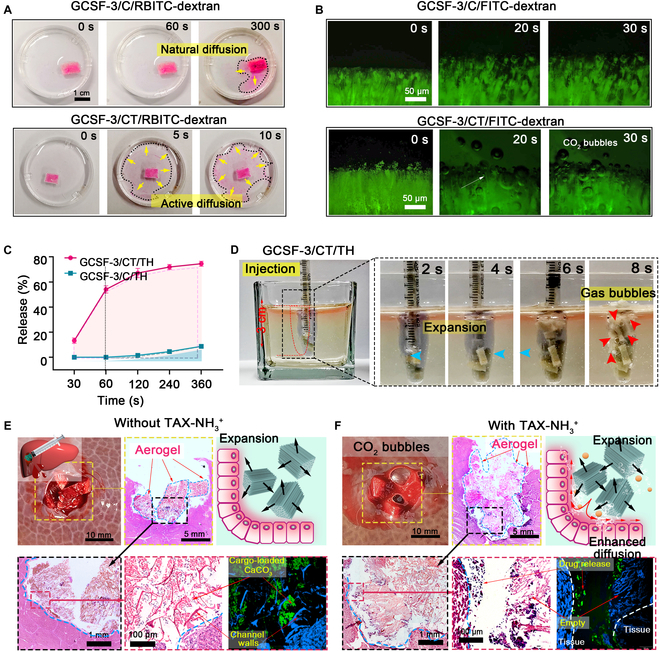
Description of payload release from aerogels via fluorescence and spectrophotometric techniques and in vitro model. (A) Time-frame images (top view) illustrating the release of RBITC-dextran from aerogels with or without TXA-NH_3_^+^. (B) Microscope image illustrating the release process of FITC-labeled dextran from GCSF-3/C/FITC-dextran or GCSF-3/CT/FITC-dextran upon contacting water. (C) Release profile of thrombin from aerogels with or without TXA-NH_3_^+^. (D) Experiment of GCSF-3/CT/TH expanding in vitro wound model that mimics massive bleeding. (E and F) Macro images and histological sections for HE staining and fluorescence staining showing the expansion and drug release of aerogels with or without TXA-NH_3_^+^ inside a perforation wound on excised pig liver.

The cargo release profiles of bombardier beetle-inspired aerogels and their potential applications in promoting cargo permeation and diffusion at bleeding sites were characterized. Before the test, thrombin (TH)-loaded CaCO_3_ and TXA-NH_3_^+^ were introduced into GCSF-3 to obtain the tested aerogel (GCSF-3/CT/TH). An aerogel containing TH-loaded CaCO_3_ (GCSF-3/C/TH) was selected for comparison. As demonstrated by the time-frame images corresponding to Movie S6 in Fig. S5A, the CO_2_ microbubbles released from the microchannel pushed the aerogel forward via a strong propulsion thrust, leading to efficient fluid convection. Fluorescein isothiocyanate (FITC)/ Rhodamine B isothiocyanate (RBITC-dextran) was employed as a model drug to replace TH to assess the cargo release behavior and visualize the release process. As shown in Fig. 6A, RBITC-dextran (red) in the GCSF-3/C/RBITC-dextran group was released slowly via natural diffusion. A schematic illustration of gas ejection-induced active release in GCSF-3/CT/TH is shown in Fig. S5B. In contrast, after the propulsion of the gas microbubbles, RBITC-dextran rapidly spread to the surroundings within 10 s, displaying a larger diffusion radius.

Similarly, fluorescence microscopy images showed that the green fluorescence in the GCSF-3/CT/FITC-dextran group rapidly diminished after adding water, and the fluorescence signal was observed in the background (Fig. 6B). In contrast, no background fluorescence was observed in the GCSF-3/C/FITC-dextran group. TH release curves over time, measured using the S-2238 assay, are shown in Fig. 6C. The results displayed that the GCSF-3/CT/TH group presented more than 50% of release within 60 s, whereas the GCSF-3/C/TH group only released less than 10% within 6 min. These results suggest that the entrapped cargo-loaded CaCO_3_/TXA-NH_3_^+^, as a built-in engine, enhanced the release of TH or fluorescent dye via active microbubble-powered delivery routes, corroborating the enhanced cargo permeation capacity of bombardier beetle-inspired aerogels.

To further evaluate the cargo release behavior of GCSF-3/CT/TH, an in vitro wound model with a conical shape mimicking a deep, narrow, non-compressible wound was fabricated. As shown in Fig. 6D, compressed GCSF-3/CT/TH in a syringe was injected into an in vitro wound model. The compressed GCSF-3/CT/TH quickly expanded to its original shape by absorbing the liquid and thoroughly filling the wound within 8 s. Additionally, large amounts of CO_2_ microbubbles (red arrows) were sprayed from the aerogels, implying a rapid release of TH within the aerogels (Movie S7). The autonomous and vigorous convection induced by CO_2_ microbubbles can further promote drug permeation within the wound. A perforation wound (15 mm in diameter and 15 mm in height) was created in the excised pig liver (Fig. 6E and F) to characterize a more realistic condition. In the GCSF-3/CT/TH group, aerogels with a fixed shape rapidly recovered their original shape to fill the wound and generate gas microbubbles after contact with blood. For the GCSF-3/C/TH group, only the expansion behavior of the aerogels was observed owing to the lack of TXA-NH_3_^+^. The tissues around the wound were examined using hematoxylin and eosin (HE) sections and fluorescent staining to characterize the distribution of the aerogels and cargo release behavior within the cavity. Figure 6E and F shows that both aerogels can expand to adapt to the wound cavity and generate mechanical compression of the wound walls. Notably, the initially filled microchannel became nearly empty in the GCSF-3/CT/TH group, and the payloads (green fluorescence) were ejected from the microchannel.

In contrast, the payloads remained in the microchannel structure of the GCSF-3/C/TH group. These results illustrate that gas microbubble-induced ejection from the microchannel greatly enhances cargo permeation and diffusion in a perforation wound. Therefore, gas-powered ejection could accelerate further penetration of payloads and overcome the insufficient adaptation of volume-expansion hemostatic aerogels. Such an approach, inspired by bombardier beetles to eject cargo and produce strong liquid convection, is essential to achieve rapid hemostasis in a timely and effective manner.

### In vitro hemostasis performance

**Fig. 7.**
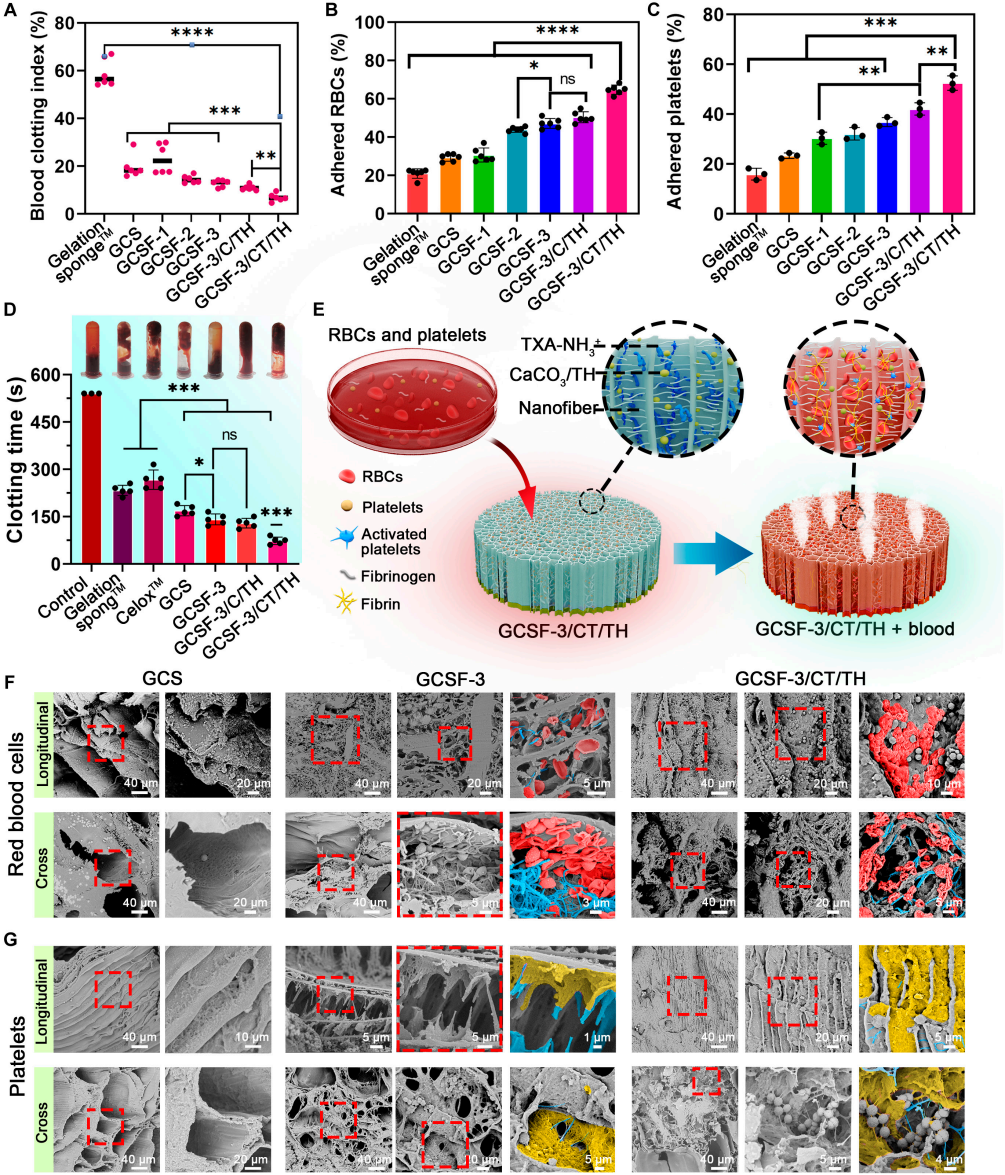
In vitro hemostatic capacity of various aerogels. (A) Blood clotting index assay. (B and C) Results of erythrocytes and platelet adhesion assays. (D) Results of the whole blood clotting test. (E) Illustration of the hemostatic mechanism of the GCSF-3/CT/TH. (F and G) SEM images illustrating the adhesion of erythrocytes and platelets on GCS, GCSF-3, and GCSF-3/CT. Red pseudocolor: erythrocytes; Yellow pseudocolor: platelets; Blue pseudocolor: fibroin nanofiber.

The blood clotting index (BCI) test was first used to evaluate the in vitro procoagulant ability of various aerogels, in which a lower BCI value indicated better coagulation capacity (Fig. 7A). The BCI values of GCS and GCSFs were significantly lower than those of the gelatin sponge. For GCS and GCSF-1, -2, and -3, the BCI value gradually decreased with increasing S-NF, reflecting a strong positive relationship between the fibrin nanofibers and coagulation ability. After introducing TH-loaded CaCO_3_ and TXA-NH_3_^+^, the BCI value of GCSF-3/CT/TH decreased to 6.87% ± 1.52%, significantly lower than other groups. Based on these results, the excellent clotting activity of GCSF-3/CT/TH can be attributed to the synergistic effects of the interwoven fibrous nanofibers and CO_2_ microbubble-induced rapid drug penetration. Erythrocyte and platelet adhesion assays were performed further to illustrate the coagulation ability of the various aerogels. As expected, the numbers of erythrocytes and platelets on GCSF-1, -2, and -3 were much higher than those on GCS and gelatin sponge (Fig. 7B and C). Higher concentrations of S-NF enhanced the adhesion of erythrocytes and platelets.

Moreover, introducing TH-loaded CaCO_3_ and TXA-NH_3_^+^ into the GCSF-3/CT/TH group increased the number of adhered erythrocytes and platelets. In the whole-blood clotting test, a trend consistent with the above results was observed in Fig. 7D. The clotting time for the control group was more than 500 s. For the positive control (gelatin sponge and Celox), the clotting time decreased to 267 ± 27.50 s and 168.6 ± 14.42 s, respectively. The GCSF-3/CT/TH group displayed superior coagulant performance, significantly reducing clotting time to 73 ± 10.3 s, approximately twice as fast as that of GCSF-3/C/TH (129 ± 13.5 s). This result indicated that TXA-NH_3_^+^ could accelerate clot formation by reacting with CaCO_3_ to generate gas microbubbles, promoting drug penetration and diffusion.

The adhesion of erythrocytes and platelets was further assessed by observing the cross-sectional and longitudinal sections of various samples using SEM (Fig. 7F and G). In the GCSF-3 group, erythrocytes and platelets adhered to the surface of the microchannel wall or were distributed in the nanofiber network, and more erythrocytes and activated platelets were observed than those in the GCS group. This result indicates that the interwoven nanofibers can concentrate blood to accelerate the aggregation of erythrocytes and platelets. In the GCSF-3/CT/TH group, the gaps between the microchannels were almost filled with erythrocytes, activated platelets, and fibrin meshwork, indicating rapid clot formation within the channels. In contrast, GCSF-3/CT/TH showed better erythrocyte/platelet adhesion than GCSF-3 or GCS alone. These observations support the results mentioned above, substantiating that GCSF-3/CT/TH exerts excellent coagulation activity in vitro via the synergistic combination of a bombardier beetle-inspired ejection system and interwoven fibrous nanofibers (Fig. 7E). These data illustrate that this novel strategy, inspired by bombardier beetles, is an effective way to achieve hemostasis in vitro and has considerable potential applicability in hemostasis.

### Biocompatibility and degradability

Ideally, hemostatic aerogels should cause negligible hemolysis and cytotoxicity during application. For the hemolysis assay, various samples were directly incubated with fresh erythrocytes. Figure S6A shows that GCS, GCSF-3, and GCSF-3/CT showed good hemocompatibility, with less than 5% hemolysis ratios. The Cell Counting Kit-8 (CCK-8) and live/dead staining kits were used for cytotoxicity testing by co-incubating L929 cells with different samples. The viability of the L929 cells in all groups increased with increasing co-incubation time (Fig. S6B). Consistently, representative fluorescence images for the live/dead staining test confirmed that GCSF-3/CT had no negative influence on L929 cell growth (Fig. S6C). A laser scanning confocal microscope (LSCM) was used to assess cell growth within the GCSF-3/CT aerogel. As shown in Fig. S6D, the 3D reconstruction of the Z-stack showed that the L929 cells could permeate into the aligned microchannels. These results demonstrate that GCSF-3/CT has good hemocompatibility and cytocompatibility.

Furthermore, the in vivo degradability was assessed using a rabbit muscle implantation model to evaluate the long-term safety of GCSF-3/CT. The commercial gelatin sponge and Celox were selected as controls. As illustrated in Fig. S7A, in the Celox group, abscesses were observed within the tissues even after 12 weeks of implantation, indicating the weak biodegradation ability of Celox. Digital photographs of the gelatin sponge and GCSF-3/CT groups showed that inflammation and abscesses had almost completely disappeared after 8 weeks. HE-stained images illustrating the degree of degradation of the different samples are shown in Fig. S7B. GCSF-3/CT changed into tiny spots and was almost completely degraded within 8 weeks, similar to gelatin sponge. However, Celox exhibited a slow degradation rate, and severe inflammation was observed in the implanted powder’s outermost layer, even in the 14th week. These results confirmed that GCSF-3/CT could serve as a biodegradable hemostatic material with no significant short- or long-term toxicity. This is because chitosan and silk fibroin are the primary raw materials for the preparation of GCSF-3/CT and are prone to hydrolysis by reaction with lysozymes [32,33]. Moreover, its porous structure endowed GCSF-3/CT with the ability to absorb a large proportion of adjacent tissue fluid. This may maintain GCSF-3/CT in a swollen state and reduce its stability, leading to a faster degradation rate.

### In vivo hemostasis performance of aerogels in various bleeding models

**Fig. 8.**
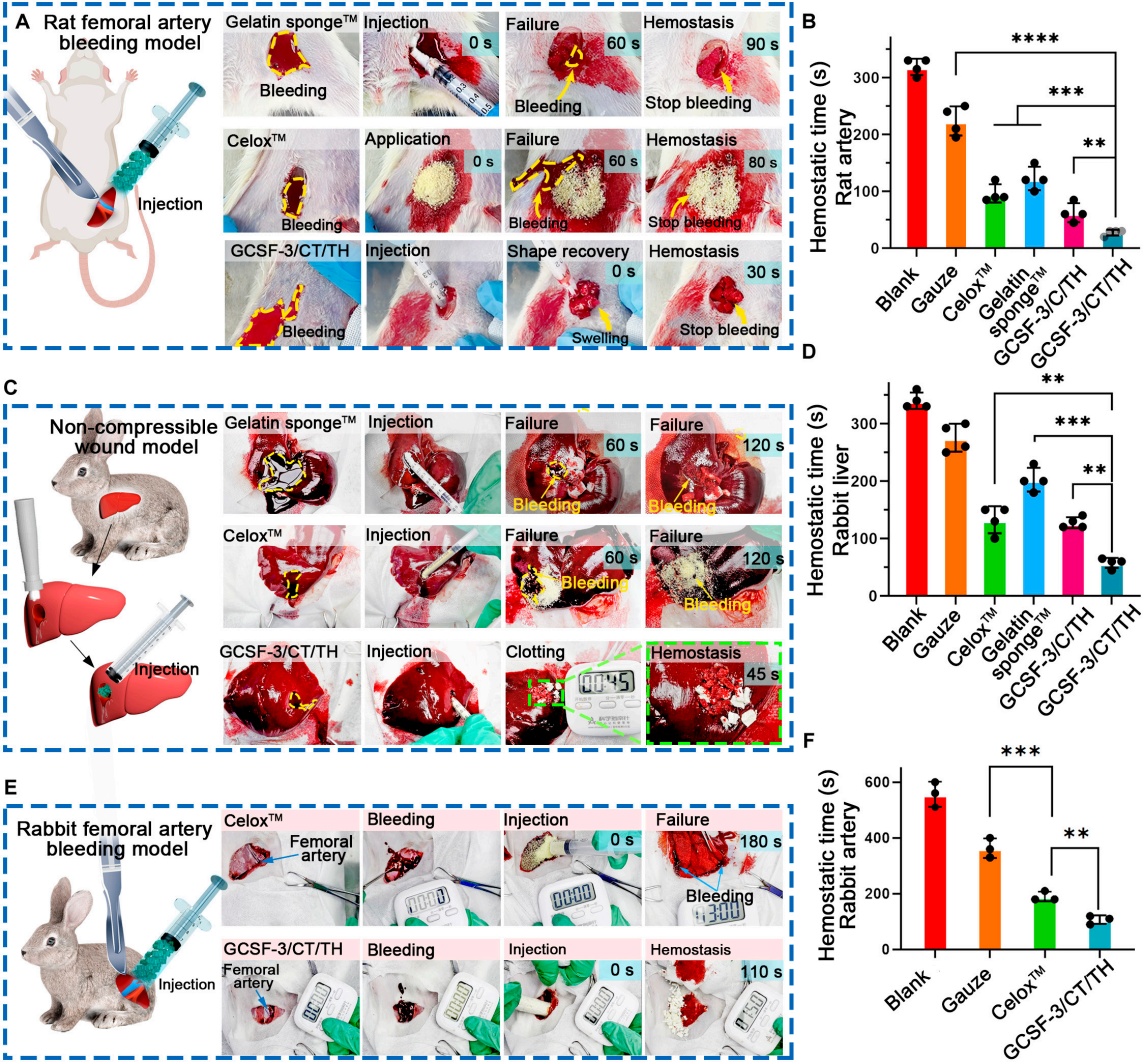
In vivo hemostatic capacity of GCSF-3/CT/TH. (A and B) Schematic illustrating the surgical procedure in rat femoral artery bleeding model, and representative optical images of the hemostatic effect of various samples and the corresponding hemostatic time. *n* = 4, **P < 0.01, ***P < 0.001, ****P < 0.0001. (C and D) Schematic and representative optical images of the hemostatic effect of various samples in a non-compressible wound model and the corresponding hemostatic time. *n* = 4, **P < 0.01, ***P < 0.001. (E and F) Schematic and representative optical images of the hemostatic effect of various samples in a rabbit femoral artery bleeding model and the corresponding hemostatic time. *n* = 3, **P < 0.01, ***P < 0.001.

The hemostatic ability of bombardier beetle-inspired aerogels was further evaluated using different in vivo bleeding models, owing to their water/blood-triggered rapid ejection performance, shape recovery ability, strong blood absorbability, and superior in vitro procoagulant activity. Gauze, a gelatin sponge, Celox, and GCSF-3/C/TH were selected for comparison. Prior to testing, GCSF-3/CT/TH and GCSF-3/C/TH with fixed shapes were cut into small pieces and loaded into syringes for further use (Fig. S8). Initially, the hemostatic activity was characterized using a rat femoral artery bleeding model (Fig. 8A). The hemostatic time for the different samples is summarized in Fig. 8B. Without treatment, the injured femoral artery showed no signs of hemostasis within 5 min. As expected, GCSF-3/CT/TH could stop bleeding within 27.4 ± 4.3 s, showing superior hemostasis activity in comparison with gauze (223.8 ± 22.2 s), gelatin sponge (122.5 ± 17.9 s), Celox (96.3 ± 13.9 s), and GCSF-3/C/TH (62.5 ± 14.4 s). The GCSF-3/C/TH group could not actively release TH because of the lack of TXA-NH_3_^+^, thus leading to a longer hemostasis time than the GCSF-3/CT/TH group. The representative digital images (Fig. 8A) showed that GCSF-3/CT/TH rapidly recovered its original shape by absorbing blood, stopping bleeding by generating pressure inside the wound, and releasing gas microbubbles to accelerate the penetration and diffusion of TH into deep bleeding sites. These findings demonstrate that the synergistic combination of a unique structure, gas-powered ejection ability, and blood-triggered shape recovery can greatly enhance hemostasis capacity.

Next, a non-compressible wound model constructed on rabbit livers was used further to assess the hemostatic properties of the different samples. As illustrated in Fig. 8C, when a small piece of shape-fixed GCSF-3/CT/TH was injected into a deep perforation wound, it rapidly absorbed blood and expanded within the bleeding cavity, forming a robust physical barrier to achieve rapid hemostasis. In comparison, gelatin sponge and Celox were quickly washed away by blood flow owing to the lack of external compression. The hemostasis times for the different samples are summarized in Fig. 8D. The GCSF-3/CT/TH-treated wound showed the shortest hemostasis time (45 ± 12.25 s), which was faster than that of gauze (270 ± 21.60 s), gelatin sponge (203.3 ± 20.54 s), and Celox (133.3 ± 25.57 s). Notably, there was no significant difference between the GCSF-3/CT/TH and GCSF-3/C/TH groups, mainly because of low blood pressure and slow blood flow in the perforated rabbit liver wound.

**Fig. 9.**
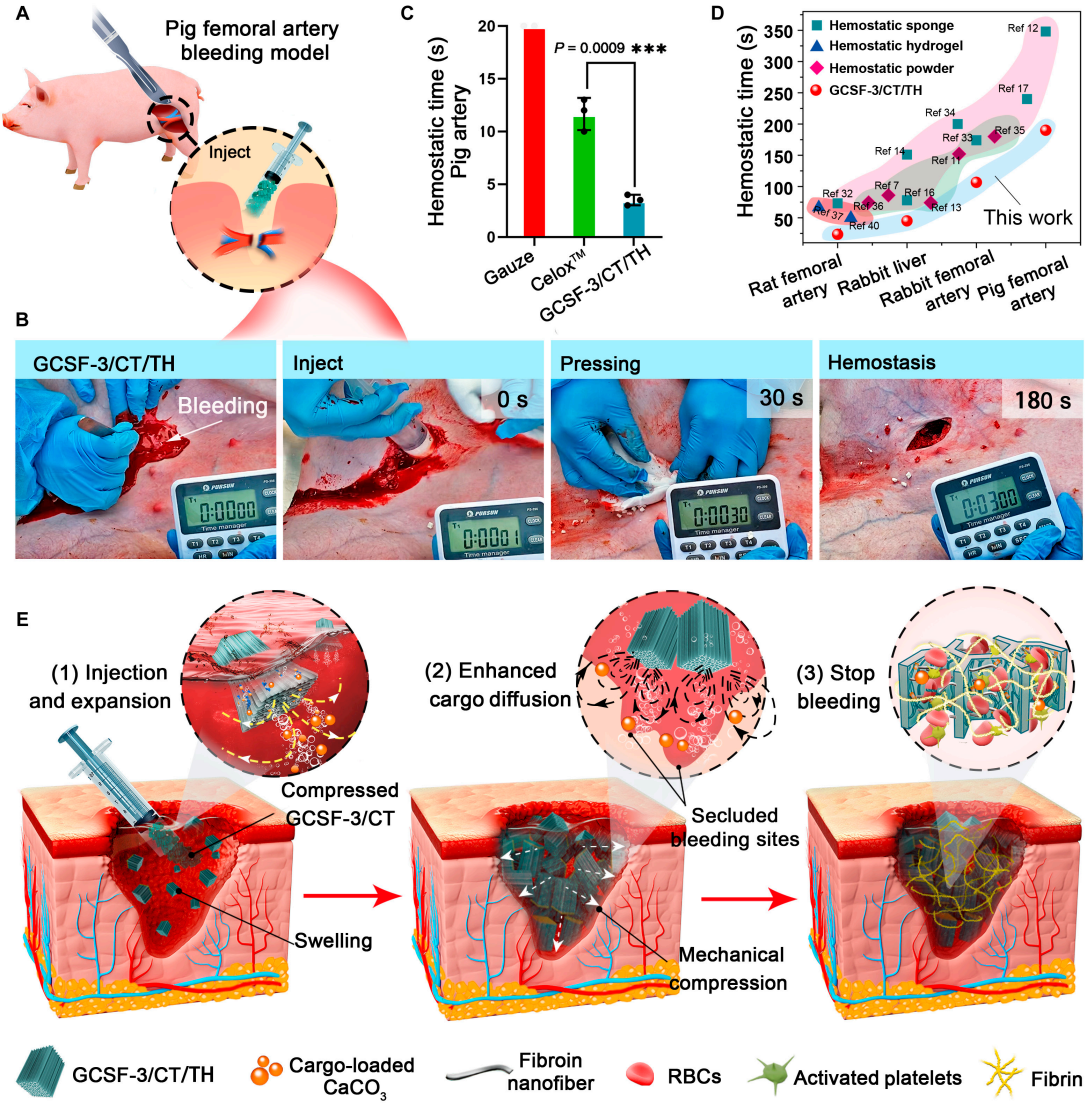
In vivo hemostatic capacity of GCSF-3/CT/TH in pig femoral artery bleeding model. (A and B) Schematic illustrating the surgical procedure in the pig femoral artery bleeding model and representative photos of the hemostatic effect of various samples. (C) The corresponding hemostatic time of various samples in the pig femoral artery bleeding model. *n* = 3, ***P < 0.001. (D) Comparison of hemostatic effect between GCSF-3/CT/TH and previously reported hemostats [11,38–43]. (E) Schematic illustrating the hemostatic mechanism of GCSF-3/CT/TH in lethal bleeding wounds.

After screening using rat femoral artery and rabbit liver bleeding models, GCSF-3/CT/TH was selected for further studies to confirm its clinical translational potential. Rabbit and pig femoral artery models of massive bleeding were established. As shown in Fig. 8E and F and Movie S8, the injection of 5 g of GCSF-3/CT/TH stopped bleeding within 106.67 ± 12.50 s, which was significantly shorter than that of Celox (190 ± 14.14 s). Consistently, representative images taken during the surgical procedure showed that many small pieces of GCSF-3/CT/TH rapidly recovered their initial shape and released pressure to seal the surface wounds, leading to rapid hemostasis and decreased blood loss. In addition, a pig femoral artery bleeding model, which has a structure similar to that of the human body, was used to explore further the hemostatic ability of GCSF-3/CT/TH (Fig. 9A and B and Movie S9). Unexpectedly, GCSF-3/CT/TH achieved rapid hemostasis within 3.5 ± 0.41 min (Fig. 9C). Celox did not control massive bleeding within 10 min. The hemostasis times for GCSF-3/CT/TH and other recently reported hemostatic materials are summarized in Fig. 9D. GCSF-3/CT/TH displayed better hemostatic performance than previously reported hemostatic sponges, hydrogels, and powders in different bleeding models. Based on these results, GCSF-3/CT/TH exhibits excellent hemostatic activity. The corresponding hemostatic mechanism can be depicted as follows (Fig. 9E): (a) After injection into the bleeding cavity, the parallel-arranged microchannel structure allows rapid blood absorption. It triggers quick recovery of its initial shape and generates pressure on the wound wall, resulting in a pressure-resistant physical barrier to block the wound. (b) The gas propulsion thrust triggers the ejection of hemostats from the microchannel, accelerating their penetration and diffusion, thereby promoting fibrin formation in the bleeding cavity. (c) The interwoven fibrous nanofibers within the microchannel concentrate the coagulation factor, accelerating the aggregation of erythrocytes and activated platelets. In summary, the synergistic combination of parallel-arranged microchannels, interwoven fibrous nanofibers, ejection performance, and shape-memory properties resulted in the remarkable hemostatic ability of bombardier beetle-inspired aerogels.

## Conclusion

Adequate hemostasis of severely bleeding wounds is extremely difficult to achieve. Unlike smaller bleeding wounds, the rapid outflow of blood from severely wounded tissues may destroy the already established blood clots that block bleeding wounds, leading to failure of hemostasis. Thus, multiple hemostatic strategies are critical for large bleeding wounds in animals and humans. We demonstrated a simple and cost-effective approach to prepare a bio-inspired aerogel that can rapidly expand to press the wound wall after contact with water/blood and generate strong microbubble-powered propulsion to accelerate the diffusion and penetration of payloads. In vitro payload release experiments demonstrated that more than 50% of the drug was released within 60 s. The results of 4 bleeding models (rat femoral artery, rabbit liver, rabbit femoral artery, and pig femoral artery) illustrated that the bombardier beetle-inspired aerogel displayed better and faster hemostatic activity than Celox and gelatin sponge. In particular, massive bleeding could be controlled within approximately 3.5 min in the pig femoral artery, which is superior to that reported for hemostatic materials. In addition, bombardier beetle-inspired aerogels showed no significant adverse effects on normal tissues and good degradability. In this regard, the multifunctional aerogel inspired by the physiological characteristics of bombardier beetles displayed excellent hemostasis for various complex wounds and is a good candidate for rapid hemostasis, paving the way for the creation of next-generation hemostatic materials.

## Methods

### Materials

Chitosan (high viscosity, >400 mPa s), tranexamic acid (TXA), gelatin, calcium chloride (CaCl_2_), and sodium carbonate (Na_2_CO_3_) were purchased from Aladdin Reagent Co. Ltd. (Shanghai, China)*.* Silk fibroin was provided by the State Key Laboratory of Silkworm Genome Biology (Southwest University, China). Dextran-fluorescein isothiocyanate (4 kDa) was purchased from Sigma-Aldrich (Shanghai, China). Tert-butanolethyl and alcohol were purchased from Chron Chemicals Co., Ltd. (Chengdu, China).

### Fabrication of the GCSF/CT

Silk fibroin nanofibers (SF-NFs), a basic structural unit of GCSF/CT, were prepared according to a previously reported electrospinning method with minor modifications [34]. Briefly, 2 g of SF was added to hexafluoroisopropanol and stirred for 2 h at 25 °C. Subsequently, the obtained solution was electrospun at a feeding speed of 0.01 ml/min using a metal needle (diameter of 0.51 mm). SF-NF was collected by an aluminum-covered roller with a receiving distance of 15 cm at a temperature of 25 ± 3 °C and a relative humidity of 55% ± 3%. SF-NF (1.0 g) was cut into square fragments (5 × 5 mm) and dispersed in tert-butanolethyl (60 ml). The mixture was then homogenized for 12 min. The obtained S-NF solution was dried by evaporation at 80 °C. For the construction of the GCSF sponge, 1 g of chitosan solution with high viscosity was dissolved in 25 ml of 1% (v v^−1^) acetic acid aqueous solution and heated at 80 °C for 30 min. S-NF was dispersed in 10 ml of deionized water and homogenized at 8,000 rpm for 3 min to form a gray slurry. The S-NF slurry was added to the chitosan solution, and 10 μl of glutaraldehyde was added. After stirring for 30 min, the mixture was poured into a square plastic mold and placed on a cryogenic copper plate connected to liquid nitrogen for directional freezing. The samples were then lyophilized for 48 h. The obtained scaffolds were put into a vacuum-drying oven at 60 °C for 4 h to accelerate the crosslinking of chitosan, S-NF, and glutaraldehyde. The S-NFs with feeding mass ratios 0%, 20%, 40%, and 60% were named GCS, GCSF-1, GCSF-2, and GCSF-3, respectively.

The CaCO_3_ microparticles were prepared by mixing equal volumes of CaCl_2_ (0.33 M) and Na_2_CO_3_ (0.33 M) for 60 s under continuous stirring (250 rpm). To synthesize fluorescent CaCO_3,_ FITC-dextran (0.5 mg/ml) was added to the CaCl_2_ solution before preparation. To prepare thrombin (TH)-loaded CaCO_3_ particles, thrombin (20 U/g) was added to a phosphate buffer saline (PBS) solution containing 10% (w v^−1^) of CaCO_3_. After incubation at 4 °C for 1 h, the precipitate was washed, collected via centrifugation, and lyophilized to obtain TH-loaded CaCO_3_. The TH loading ratio and CaCO_3_ loading content were approximately 25% and 5 U/g. Protonated tranexamic acid (TXA-NH_3_^+^) was prepared as described in a previous study [13]. A 0.5 M tranexamic acid aqueous solution was prepared, and hydrochloric acid was used to adjust the pH to 4.0. The TXA-NH_3_^+^ powder was obtained by freeze-drying.

To fabricate the brachinine-inspired aerogels, gelatin solution (4% w v^−1^) at 40 °C was infiltrated into one side of GCSF aerogels in a radial direction and allowed to cool and solidify to cap the pores. Then, 0.7 g of the composed powder (CaCO_3_ or CaCO_3_/TH) physically mixed with TXA-NH_3_^+^ at a molar ratio of 1:1 (CaCO_3_:TXA-NH_3_^+^) was absorbed into the microchannel of the GCSF aerogels (4 × 4 × 0.8 mm) via vacuum loading. The excess powder was removed using a nitrogen purge. The aerogel loaded with CaCO_3_ and TXA-NH_3_^+^ was called GCSF/CT.

### Characterization

The Fourier transform infrared spectrometer (FTIR) spectra of chitosan, S-NF, and GCSF aerogels were measured at 400 to 4,000 cm^−1^ using a Bruker Alpha spectrometer (Bruker TensorII, Germany). The hydrophilicity and hemophilicity of the GCS, GCSF, and GCSF/CT were evaluated using a contact-angle goniometer (Dataphysics, Germany). A scanning electron microscope (Phenom Pro, Netherlands) equipped with an energy-dispersive x-ray spectroscopy detector was used to record the morphology of GCS, GCSF, and GCSF/CT. The average pore size was calculated using the Nanomeasure software. Thermogravimetric spectra of GCSF and GCSF/CT were collected by TGA Q50 (USA) (20 °C/min, air atmosphere). The 3D microstructure and porosity were tested using a micro-CT (Aoying, AX-2000, China).

### Ejection performance of GCSF/CT

A high-speed camera (Phantom VEO 1030, USA) was used to capture videos of the ejection performance of GCSF/CT. For the test, a cylinder GCSF/CT (height of 4 mm and diameter of 1.5 mm) was thrown into the petri dish containing 2 ml of Tween-20 aqueous solution (0.1%). A high-speed camera was placed on the petri dish to record the ejection trajectory. Further, 500 μl of 0.1% Tween-20 aqueous solution was dropped on a slice of GCSF/CT (0.2 mm height × 5 mm width × 10 mm length), and the ejection process was recorded using an inverted optical microscope (Olympus ix73, Japan).

A digital fluid simulation validated the ejection performance of GCSF/CT. The ejection process was modeled using COMSOL Multiphysics software. To simplify the simulation process, the microchannel size of the GCSF/CT was 40 μm. The temperature, working pressure, and dynamic viscosity of the liquid were 37 °C, 1 atm, and 5.63 × 10^−3^ kg m^−1^ s^−1^, respectively. The initial velocity of microparticles launched from the microchannel was 0.027 m s^−1^.

### Characterization of drug release

GCSF loaded with CaCO_3_/TH and TXA-NH_3_^+^ (GCSF/CT/TH) was soaked in normal saline (pH 7.4). The extract was collected at 30, 60, 120, 240, and 360 s. TH released from the GCSF/CT/TH was measured using a chromogenic substrate assay (S-2238). TH release from GCSF loaded with CaCO_3_/TH (GCSF/C/TH) was also evaluated as a control. FITC-dextran was used as a model drug to replace TH to visualize the drug release process. FITC-dextran release from the aerogels (GCSF/CT/FITC-dextran or GCSF/C/FITC-dextran) was directly observed using an inverted optical microscope.

### In vitro drug penetration model

Fresh pig livers were purchased from a local market and used to determine the drug penetration capability of the aerogels. Initially, a cylindrical wound (15 mm in diameter and 15 mm in height) was created on the pig liver, and 3 ml of fresh pig blood was added to form a blood cavity. Small pieces (0.1 g) of the aerogel (2 × 2 × 5 mm) were loaded into a syringe (1 ml) and injected into the blood cavity. The wound was separated into frozen sections (20 μm) to confirm ejection performance and drug penetration inside the cavity. HE and immunofluorescence histochemical staining were conducted and evaluated using an optical microscope.

### Mechanical performance and shape-recovery test

The compression properties of the aerogels (10 mm × 10 mm × 10 mm) before and after water/blood absorption were evaluated using a universal testing machine (MTS-E44, China). The compressive strain was fixed at 80%. The compression and recovery of aerogels after water absorption were evaluated using cyclic compression tests.

The aerogels were first compressed in a shape-recovery test to achieve shape fixation, and the maximum fixation ratio was calculated. The shape-recovery time and recovery ratio in water and blood were recorded using a video camera. The absorption capacity of water/blood was also investigated according to the weight change before and after contact with water/blood for a specified time. The absorption capacity of GCSF/CT was determined using the COMSOL theoretical model. An aerogel microchannel with a diameter of 40 μm was used to simplify the simulation. Water served as a liquid matrix with a viscosity of 1.0 × 10^−3^ kg m^−1^ s^−1^. The temperature and working pressure were 37 °C and 1 atm, respectively.

### In vitro hemostasis

The hemostatic activity of the aerogels was evaluated using a BCI and whole blood coagulation assay [35]. For the BCI test, 100 μl of fresh anticoagulant rabbit blood was dropped onto the aerogels (10 × 10 × 10 mm), and 10 μl of CaCl_2_ (0.2 M) was added immediately. After incubation at 37 °C for 5 min, 30 ml of deionized water was added, and the mixture solution was incubated at 37 °C for another 15 min. The OD_540_ of the supernatant from each sample was measured using a ultraviolet-visible and near-infrared spectrophotometer (UV–vis–NIR). BCI was calculated using the following equation:

$$\mathrm{BCI}\;\left(\%\right)=\frac{{\mathrm{OD}}_{\text{control}}-{\mathrm{OD}}_{\text{sample}}}{{\mathrm{OD}}_{\text{control}}}\times 100\%$$

where OD_control_ is the absorbance of the group without aerogel treatment and OD_sample_ is the absorbance of the supernatant in the tested aerogels.

Blood coagulation assays were performed as previously described [36]. Briefly, 0.05 g of different samples was added to 2 ml of fresh anticoagulant rabbit blood, and 80 μl of CaCl_2_ (0.2 M) was added immediately. The clotting time was recorded when the blood lost its fluidity. The experiments mentioned above were carried out at 37 °C.

### RBC and platelet adhesion assays

The interaction between GCSF/CT and red blood cell (RBC) or platelet adhesion was investigated according to a previous study [37]. Before the test, RBCs and platelet-rich plasma (PRP) were separated by centrifugation (1,500 rpm, 10 min). For RBC adhesion, 100 μl of RBCs was added to the surface of various aerogels (10 × 10 × 10 mm) and incubated at 37 °C for 1 h. Subsequently, aerogels were rinsed with PBS solution and placed into 4 ml of deionized water to lyse adhered RBCs. RBC adhesion in different groups was evaluated based on the OD_540_ value of the supernatant.

For the platelet adhesion experiment, various aerogels (10 × 10 × 10 mm) containing 100 μl of PRP were incubated at 37 °C for 1 h. Next, 4 ml of Triton X-100 solution was used to lyse adhered platelets, releasing the lactate dehydrogenase (LDH) enzyme. Subsequently, an LDH kit (Biyuntian, China) was used to measure the LDH concentration. The percentage of platelet adhesion was calculated according to the manufacturer’s instructions.

After treatment with RBCs/PRPs, various aerogels were fixed with 2.5% of glutaraldehyde and dehydrated by gradient alcohol (50%, 60%, 70%, 80%, 90%, and 100%) to observe the interaction between various aerogels and RBCs/platelets. The cross-sectional and longitudinal sections of the various aerogels were observed using SEM.

### Hemocompatibility and cytocompatibility tests

The hemocompatibility of the various aerogels was investigated by measuring the hemoglobin concentration released from the treated RBCs. Briefly, RBCs were separated from the anticoagulated rabbit blood by centrifugation (1,500 rpm, 10 min) and diluted to 4% (v/v) with normal saline. Then, various aerogels (10 × 10 × 10 mm) were placed in a centrifuge tube containing 3 ml of the RBC suspension. The supernatant of each group was collected, and its absorbance at 540 nm was measured using a UV–vis–NIR absorbance spectrometer after incubation for 2 h at 37 °C. The hemolysis ratio (HR) was calculated based on the following equation:

$$\mathrm{HR}\;\left(\%\right)=\frac{{\mathrm{OD}}_{\text{sample}}-{\mathrm{OD}}_{\text{positive}}}{{\mathrm{OD}}_{\text{negative}}-{\mathrm{OD}}_{\text{positive}}}\times 100\%$$

where OD_sample_, OD_positive_, and OD_negative_ represent the absorbances of the supernatant in the tested samples, normal saline, and deionized water groups, respectively.

The CCK-8 and live/dead staining kits were used for cytocompatibility tests. Before the test, the aerogels were soaked in alcohol (75%) for 20 min and then washed with normal saline for sterilization. A suspension of L929 cells was added to a 96-well plate (10^4^/well) and incubated for 4 h at 37 °C to make cells adhere to the bottom of the well plate. Subsequently, sterilized aerogels (0.2 mm height, 2 mm width, and 2 mm length) were added to the cell suspension. At specific time points (24, 48, and 72 h), the cell viability was evaluated using a CCK-8 kit. The CCK-8 agent was directly added into 96-well plates, and then the OD_450_ was tested using a microplate photometer (Multiskan FC, USA) after incubation for 2 h at 37 °C. The cell viability was calculated as follows:

$$\text{Cell viability}\;\left(\%\right)=\frac{{\mathrm{OD}}_{\text{sample}}}{{\mathrm{OD}}_{\text{control}}}\times 100\%$$

Live/dead cells were stained with calcein-AM and propidium iodide dyes and visualized under a microscope. Furthermore, 200 μl of L929 cell suspension (10^4^ cells) was dropped on the surface of the GCSF/CT (0.2 mm height, × 5 mm width, × 5 mm length). The cells were placed in a 24 well-plate containing 1 ml of fresh medium. After incubation for 48 h at 37 °C, the aerogels were observed under an LSCM (FV3000RS, OLYMPUS, Japan).

### In vivo degradation

All animal studies complied with the protocols approved by the National Center of Animal Science Experimental Teaching of the Southwest University of China (accreditation number: SWU_LAC-20210481). A muscle implantation model was constructed to investigate the inability of GCSF/CT in vivo. New Zealand white rabbits (male, approximately 2 kg, 2 months old) obtained from the local Animal Laboratory Center were used in this study. The fur on the backs of the rabbits was shaved after anesthesia. Subsequently, a 1-cm incision was made on the back muscle, and 0.1 g of sterilized GCSF/CT was stuffed inside the wound via sutures. After 2, 4, 6, 8, and 10 weeks of feeding, the back tissue around the incision was excised and immersed in formalin (10%) for HE staining. Celox (USA) was used as a control.

### In vivo hemostasis performance analysis

The hemostatic performance of GCSF-3/CT/TH was evaluated using rabbit liver trauma, rat femoral artery, rabbit femoral artery, and pig femoral artery bleeding models. Gauze, Celox, Gelatin, and GCSF-3/CT were used as controls. All animal experiments complied with protocols approved by the National Center of Animal Science Experimental Teaching of the Southwest University of China (accreditation number of the laboratory/investigator: SWU_LAC_202110481). All animal bleeding model experiments were conducted in triplicate for each group.

Rat femoral artery bleeding model: SD rats (male, approximately 250 g) were anesthetized by injecting pentobarbital sodium (40 mg/kg). The rats were fixed on the operating table, and the inner thigh fur was shaved. Subsequently, an incision (1 to 2 cm in length and 0.3 to 0.5 cm in depth) was created in the femoral artery to induce bleeding. Compressed small pieces (0.5 g) of GCSF-3/CT/TH loaded into a 1-ml syringe were immediately injected into the bleeding cavity. The process was recorded using a video camera.

Rabbit liver trauma model: New Zealand white rabbits (male, approximately 2 kg, 2 months old) were anesthetized, and their livers were removed and placed on the surface of gauze. Next, a cylindrical wound with a diameter of 10 mm and a depth of 5 mm was made on the liver. Small compressed pieces (0.5 g) of GCSF-3/CT/TH were immediately placed in the wound. A similar procedure was performed according to the method mentioned above for the other groups. The hemostasis and clotting times were recorded.

Rabbit femoral artery massive bleeding model: The procedures were performed according to the method described above for the rat femoral artery bleeding model. The femoral arteries of the rabbits were transected to form an incision (5 cm in length and 2 cm in depth), and 5 g of small compressed pieces of GCSF-3/CT/TH was immediately injected into the wound cavity to stop the bleeding. The hemostasis and clotting times were recorded.

Pig femoral artery massive bleeding model: A pig obtained from a local market (male, 40 kg) was anesthetized using a respiratory anesthesia machine, and breath and heart rates were monitored. The femoral artery in the inner thigh was transected entirely to form a deep incision (7 to 10 cm in length and 5 to 7 cm in depth) to induce massive bleeding. Subsequently, small compressed pieces (20 g) of GCSF/CT/TH were injected into the wound cavity. The hemostasis and clotting times were recorded.

## Data Avialability

The data used to support the findings of this study are available from the corresponding author upon request.
