# Supplementary material for: Bioinspired Hemostatic Strategy via Pulse Ejections for Severe Bleeding Wounds
Source: Research (Wash D C). 2023 May 22;6:0150. doi: 10.34133/research.0150 (PMC10202099; doi:10.34133/research.0150)
Supplement: Supplementary 1 — Figs. S1 to S8 [file research.0150.f1.docx]

Supplementary Materials for

**Bioinspired hemostatic strategy via pulse ejections for severe bleeding wounds**

*Bitao Lu* ^1^*, Enling Hu* ^1,2^*, Weiwei Ding*^3^*, Wenyi Wang*^4^*, Ruiqi Xie* ^1,2^*, Kun Yu* ^1,2^*, Fei Lu* ^1,2^*,* *Guangqian Lan*^1,2^**, Fangyin Dai* ^1,2^**^[[1]](#footnote-1)^*

^1^State Key Laboratory of Silkworm Genome Biology, College of Sericulture, Textile and Biomass Sciences, Southwest University, Chongqing 400715, China

^2^Chongqing Engineering Research Center of Biomaterial Fiber and Modern Textile, Chongqing 400715, China

^3^ Division of Trauma and Surgical Intensive Care Unit, Research Institute of General Surgery, Jinling Hospital, Medical School of Nanjing University, Nanjing, 210002 Jiangsu Province, China

^4^ Department of Applied Biology and Chemical Technology, The Hong Kong Polytechnic University, Hong Kong, China.

*Corresponding author. Email: *Fangyin Dai.* fydai@swu.edu.cn; *Guangqian Lan.* j070218@swu.edu.cn

**This PDF file includes:**

1. Supplementary Figures

3. Supplementary Movie Description


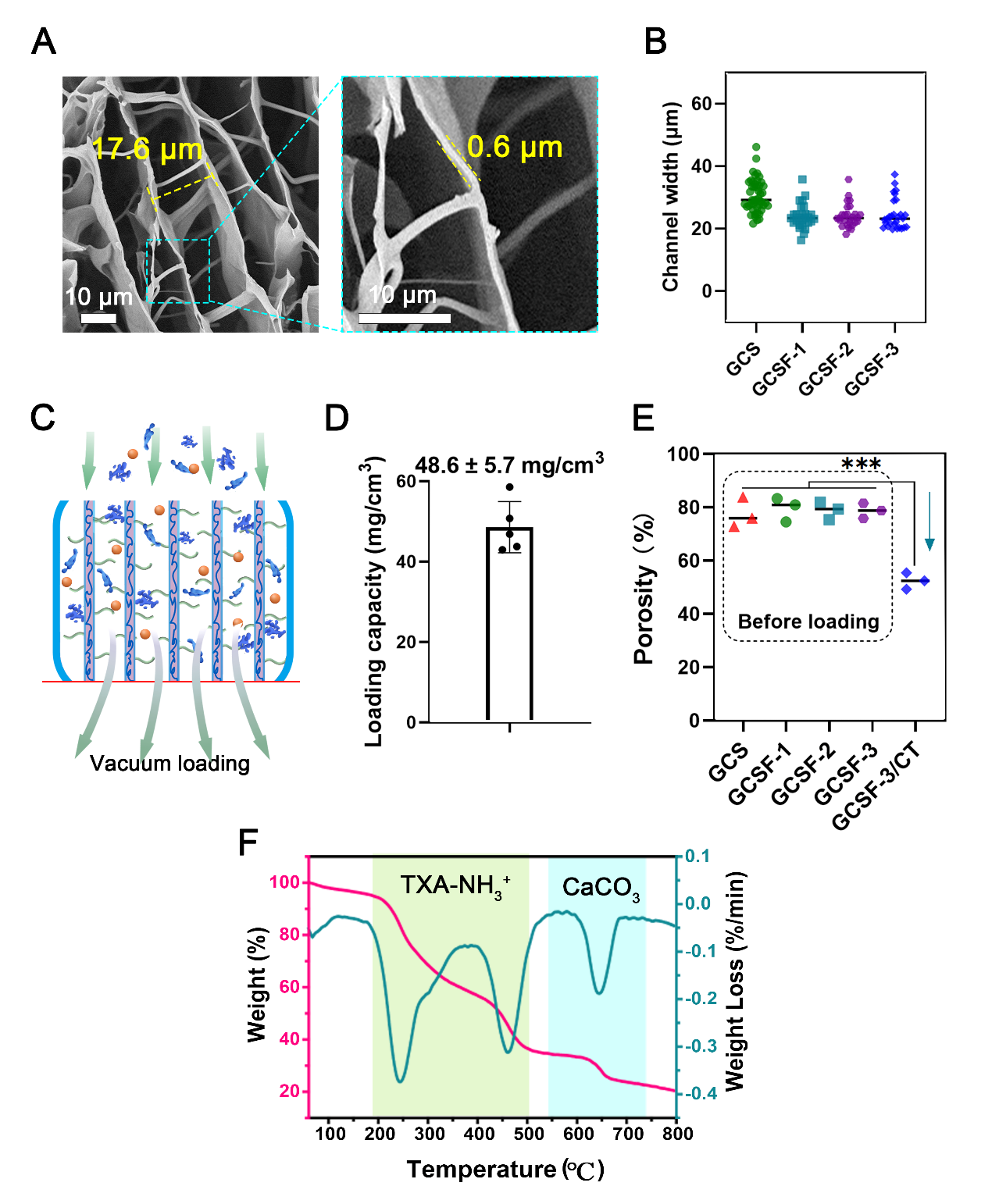


Supplementary Fig. 1 A) SEM images viewing channel width and the thickness of channel wall. B) Channel width of different aerogels. C) Schematic illustration of the vacuum loading process. D) The cargo loading capacity of GCSF-3/CT. E) The porosity of GCS, GCSF-1/2/3 and GCSF-3/CT. F）TG and DTG curves of GCSF-3/CT.


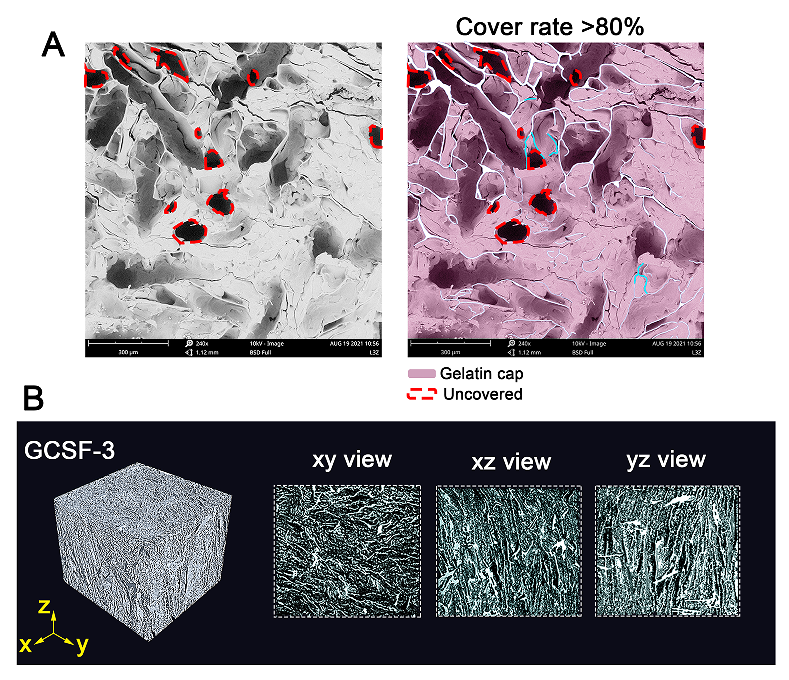


Supplementary Fig. 2 A) Bottom view of images of GCSF-3/CT (pink pseudocolor represents gelatin cap, white pseudocolor represents channel walls, and the dotted line represents the uncovered area). B) Micro-CT images of GCSF-3.


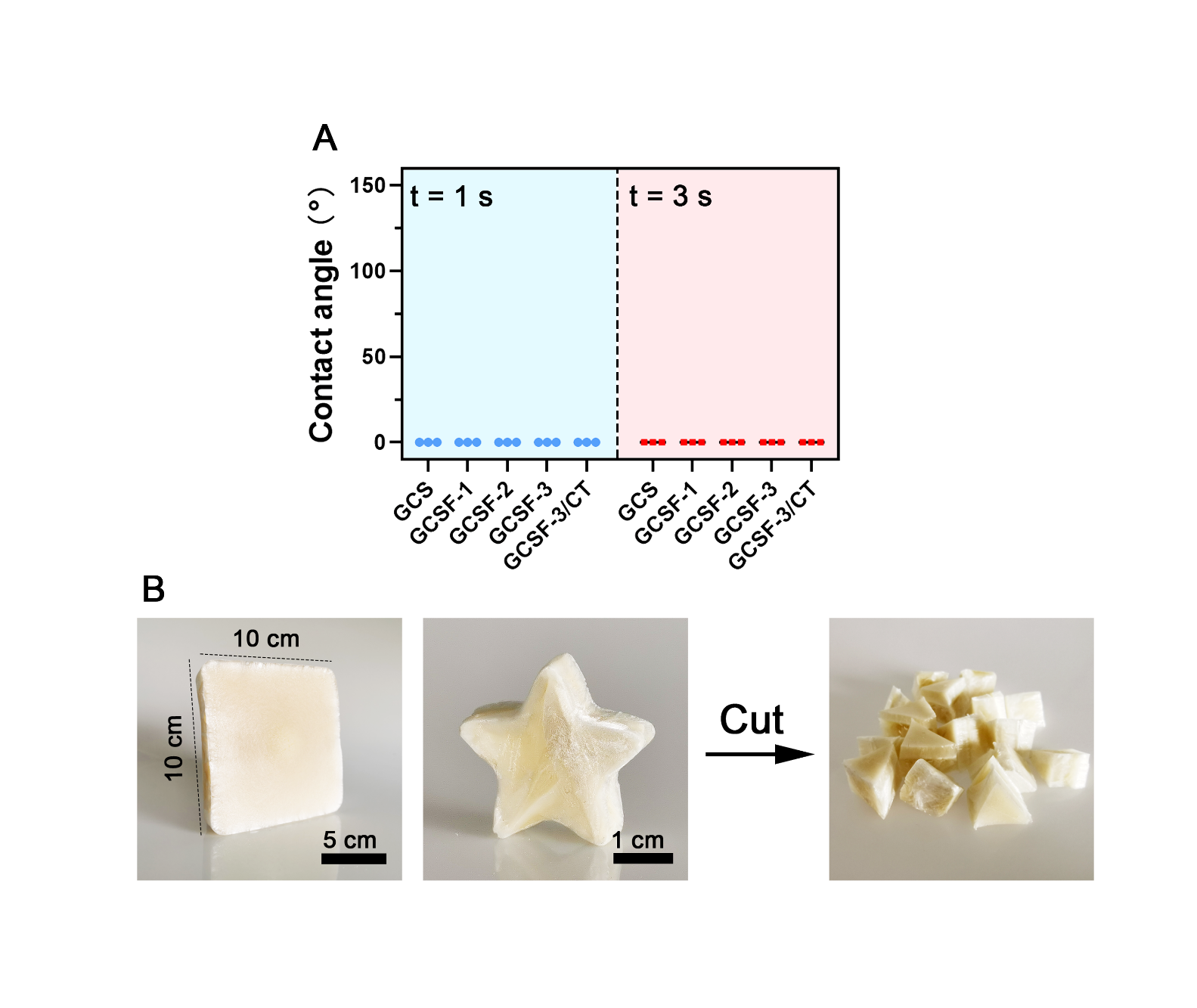


Supplementary Fig. 3 A) The hydrophilicity and hemophilicity of different aerogels. B) Macro photographs of GCSF-3/CT with different shape.


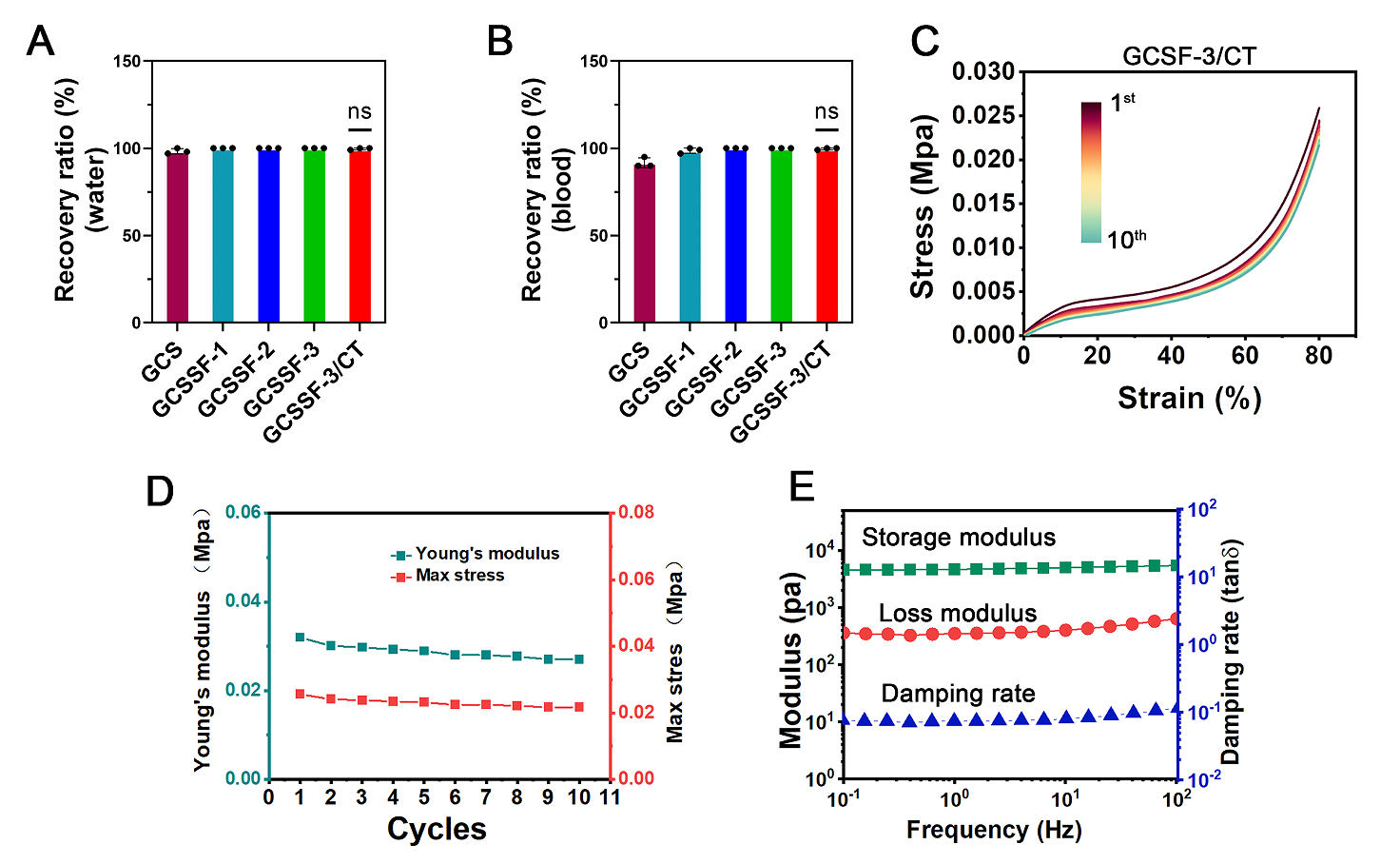


Supplementary Fig. 4 A, B) Shape-recovery ratio of compressed aerogels upon contacting water and blood. C) Cyclic compressive stress-strain curve of GCSF-3/CT. D) The change of

maximum stress and Young’s modulus of GCSF-3/CT during the cyclic compressive test. E) The change of storage modulus, loss modulus and damping rate of GCSF-3/CT over the angular frequency range from 0.1 to 100 Hz.


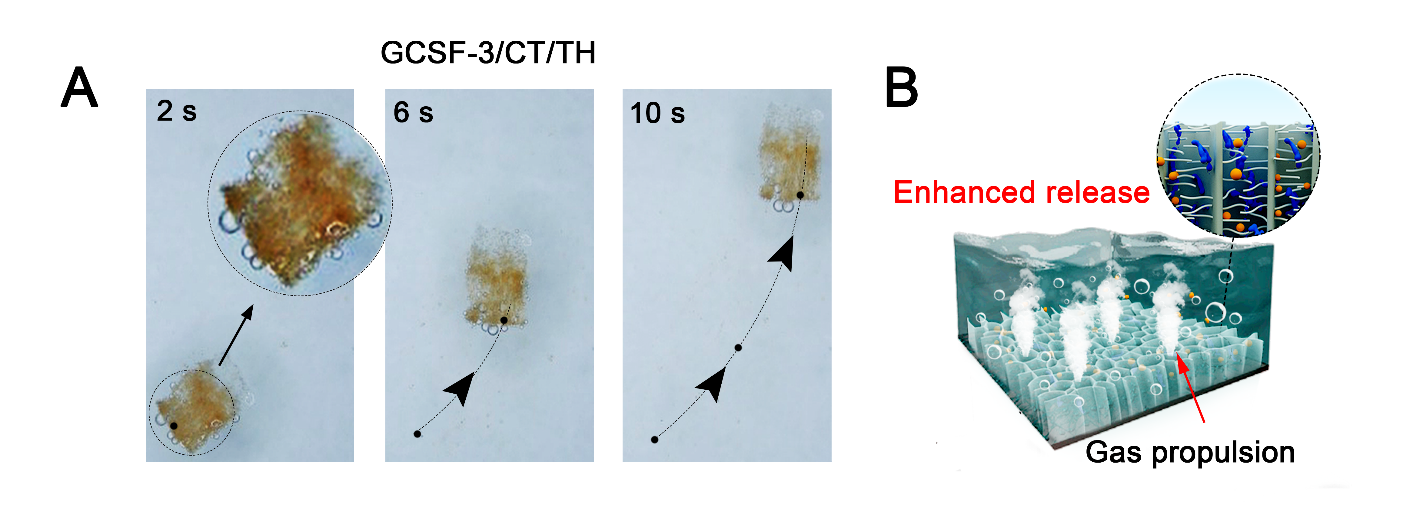


Supplementary Fig. 5 A) Time-frame images showing the propulsion of GCSF-3/CT/TH after contacting water via gas thrust. B) Schematic of gas propulsion leading to enhanced drug release from GCSF-3/CT/TH.


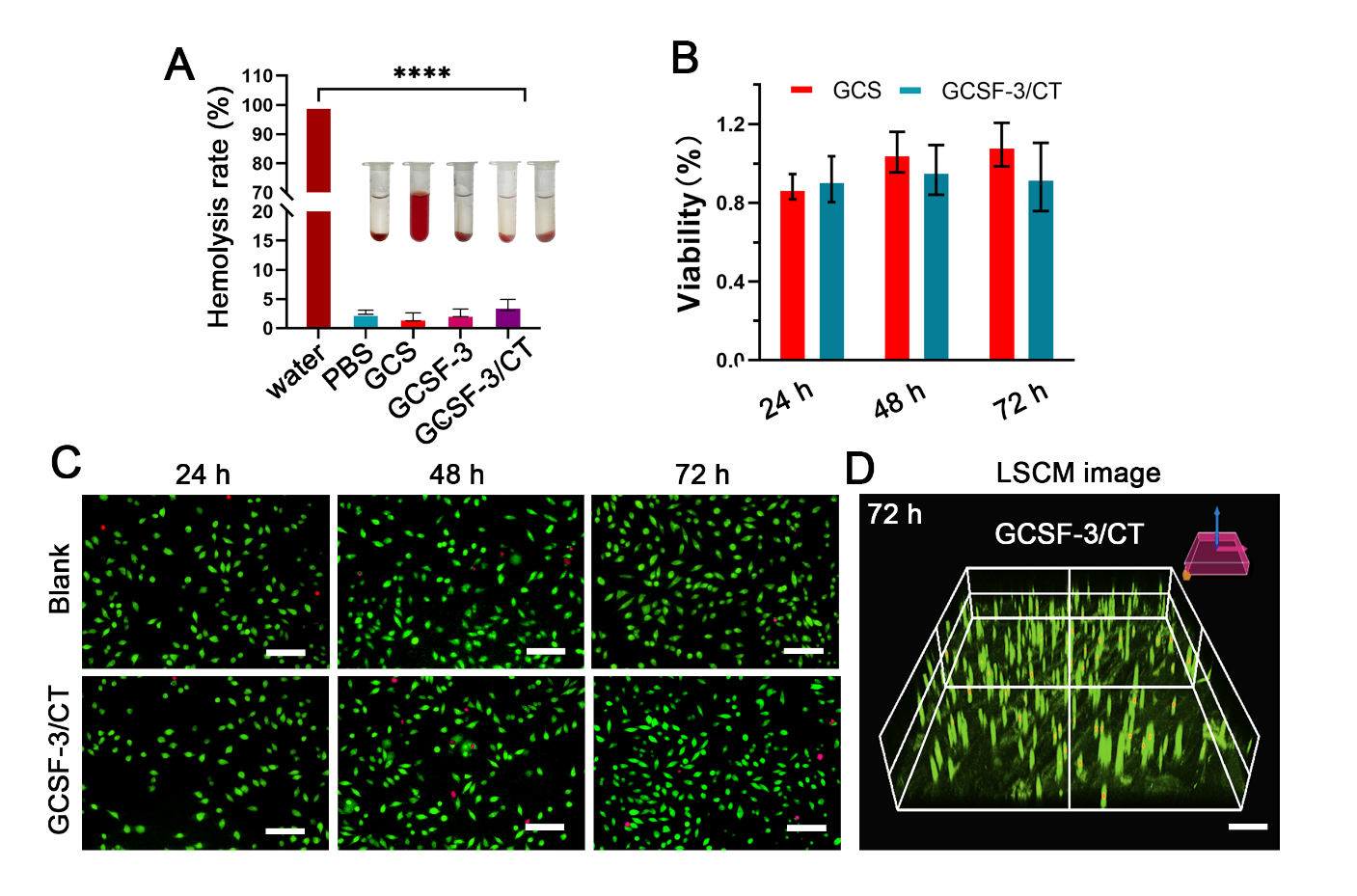


Supplementary Fig. 6 A) Hemolysis rate of GCS, GCSF-3 and GCSF-3/CT. B) The viability of L929 cells after incubation with GCS and GCSF-3/CT for 24, 48 and 72 h. C) Representative Live/Dead staining images of L929 cells after incubation with GCSF-3/CT for 24, 48 and 72 h. D) Fluorescence micrographs showing distribution of Live/Dead cells in GCSF-3/CT structure after incubation for 72 h.


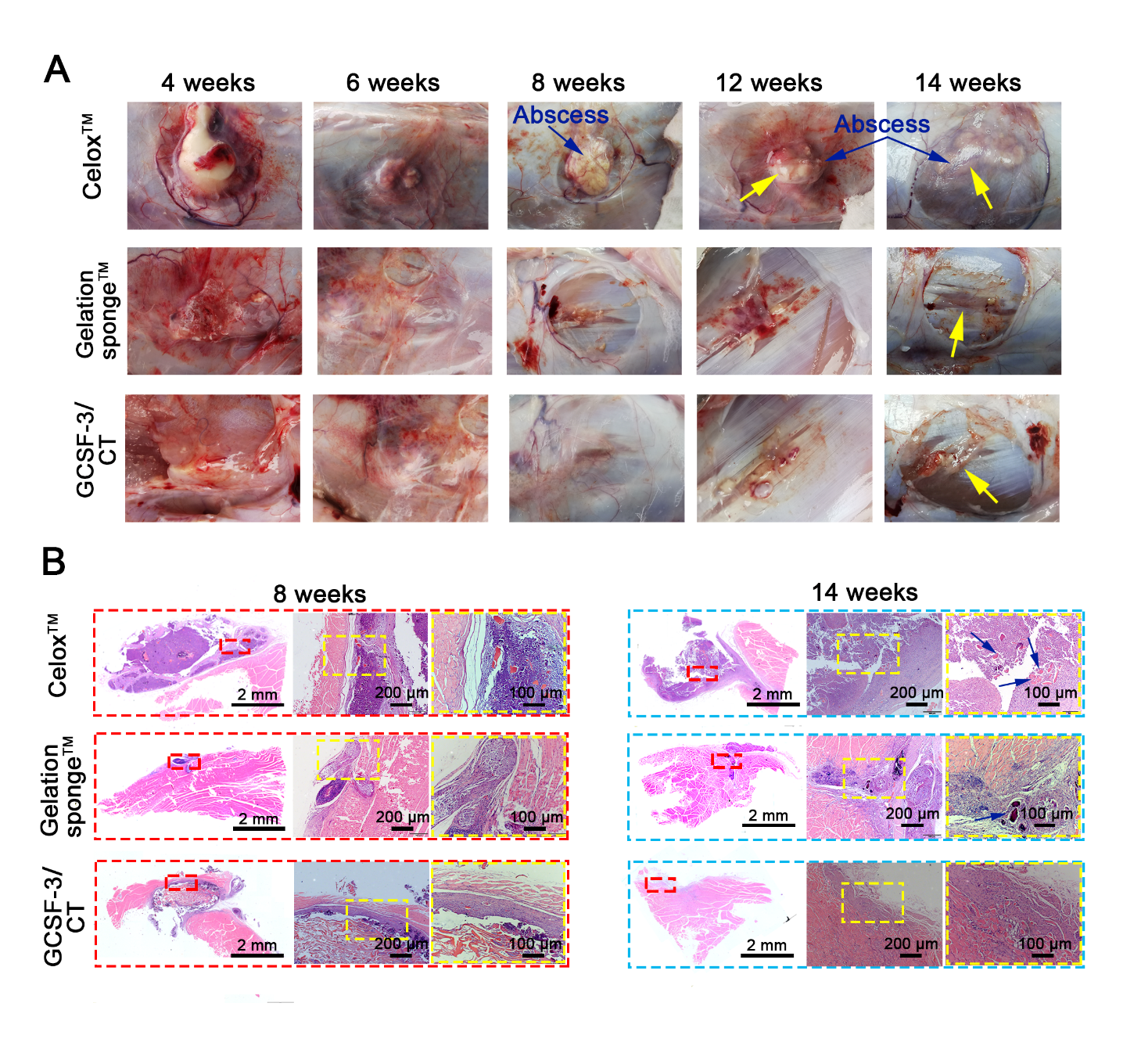


Supplementary Fig. 7 A) Degradation performance of GCSF-3/CT, gelatin sponge^TM^ and Celox^TM^ in rabbit muscle implantation models. The wound site is marked with yellow arrows; B) Typical H&E staining images of liver and muscle after implantation for 8 and 14 weeks. The blue arrows indicate the residues.


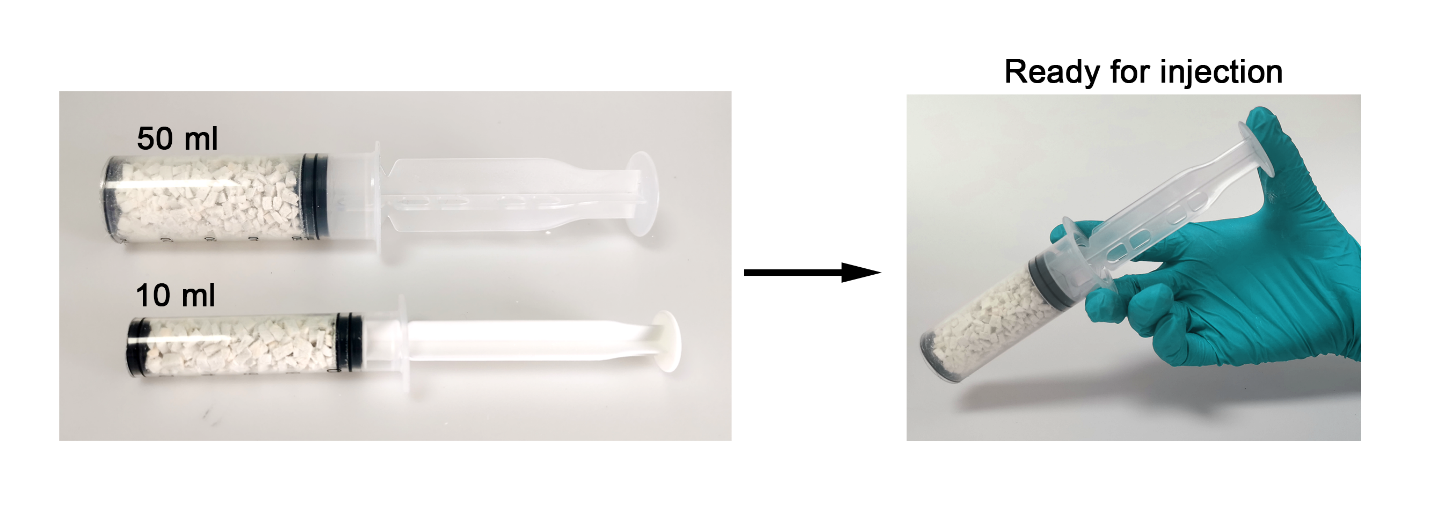


Supplementary Fig. 8 The home-made device loading with GCSF-3/CT/TH.

**2. Supplementary Movie Description**

**Supplementary Movie 1**

Time-lapse frames (0-5 s) using a high-speed camera sketching the water-triggered ejection process.

**Supplementary Movie 2**

Time-lapse frames (0-30 s) captured by microscope illustrating the gas-powered ejection performance in microchannels of bio-inspired aerogel.

**Supplementary Movie 3**

Video of the ejection of cargo-loaded CaCO_3_ under the propulsion of expanded microbubbles.

**Supplementary Movie 4**

The morphology change of a single microbubble during the ejection process.

**Supplementary Movie 5**

COMSOL simulation of the ejection behavior and fluid convection generated from GCSF-3/CT/TH after contact with water.

**Supplementary Movie 6**

Water-triggered actuation of GCSF-3/CT/TH under propulsion of microbubbles.

**Supplementary Movie 7**

The rapid expansion of GCSF-3/CT/TH in vitro wound model after injection.

**Supplementary Movie 8**

Hemostasis of the GCSF-3/CT/TH in lethal rabbit femoral artery hemorrhage model.

**Supplementary Movie 9**

Hemostasis of the GCSF-3/CT/TH in lethal pig femoral artery hemorrhage model.

1. **Fangyin Dai.* fydai@swu.edu.cn;

   **Guangqian Lan.* j070218@swu.edu.cn; [↑](#footnote-ref-1)
